# Structural basis for neutralization of hepatitis A virus informs a rational design of highly potent inhibitors

**DOI:** 10.1371/journal.pbio.3000229

**Published:** 2019-04-30

**Authors:** Lei Cao, Pi Liu, Pan Yang, Qiang Gao, Hong Li, Yao Sun, Ling Zhu, Jianping Lin, Dan Su, Zihe Rao, Xiangxi Wang

**Affiliations:** 1 CAS Key Laboratory of Infection and Immunity, CAS Centre for Excellence in Biomacromolecules, Institute of Biophysics, Chinese Academy of Sciences, Beijing, China; 2 State Key Laboratory of Biotherapy, West China Hospital, Sichuan University, Collaborative Innovation Center for Biotherapy, Chengdu, China; 3 National Laboratory of Macromolecules, Institute of Biophysics, Chinese Academy of Sciences, Beijing, China; 4 Biodesign Center, Tianjin Institute of Industrial Biotechnology, Chinese Academy of Sciences, Tianjin, China; 5 Sinovac Biotech Co., Ltd., Beijing, China; 6 Tianjin International Biomedical Joint Research Institute, Tianjin, China; 7 Laboratory of Structural Biology, School of Medicine, Tsinghua University, Beijing, China; National Institute of Allergy and Infectious Diseases, UNITED STATES

## Abstract

Hepatitis A virus (HAV), an enigmatic and ancient pathogen, is a major causative agent of acute viral hepatitis worldwide. Although there are effective vaccines, antivirals against HAV infection are still required, especially during fulminant hepatitis outbreaks. A more in-depth understanding of the antigenic characteristics of HAV and the mechanisms of neutralization could aid in the development of rationally designed antiviral drugs targeting HAV. In this paper, 4 new antibodies—F4, F6, F7, and F9—are reported that potently neutralize HAV at 50% neutralizing concentration values (neut_50_) ranging from 0.1 nM to 0.85 nM. High-resolution cryo-electron microscopy (cryo-EM) structures of HAV bound to F4, F6, F7, and F9, together with results of our previous studies on R10 fragment of antigen binding (Fab)-HAV complex, shed light on the locations and nature of the epitopes recognized by the 5 neutralizing monoclonal antibodies (NAbs). All the epitopes locate within the same patch and are highly conserved. The key structure-activity correlates based on the antigenic sites have been established. Based on the structural data of the single conserved antigenic site and key structure-activity correlates, one promising drug candidate named golvatinib was identified by in silico docking studies. Cell-based antiviral assays confirmed that golvatinib is capable of blocking HAV infection effectively with a 50% inhibitory concentration (IC_50_) of approximately 1 μM. These results suggest that the single conserved antigenic site from complete HAV capsid is a good antiviral target and that golvatinib could function as a lead compound for anti-HAV drug development.

## Abstract 2

Structures of hepatitis A virus in complex with five neutralizing antibodies reveal a single conserved antigenic site and pinpoint key structure-activity correlates, allowing in silico screening to identify a potent candidate inhibitor drug, golvatinib.

## Author summary

Hepatitis A virus (HAV) is a unique, hepatotropic human picornavirus that infects approximately 1.5 million people annually and continues to cause mortality despite a successful vaccine. There are no licensed therapeutic drugs to date. Better knowledge of HAV antigenic features and neutralizing mechanisms will facilitate the development of HAV-targeting antiviral drugs. In this study, we report 4 potent HAV-specific neutralizing monoclonal antibodies (NAbs), together with our previous reported R10, that efficiently inhibit HAV infection by blocking attachment to the host cell. All 5 epitopes are located within the same patch and are highly conserved across 6 genotypes of human HAV, which suggests a single antigenic site for HAV, highlighting a prime target for structure-based drug design. Analysis of complexes with the 5 NAbs with varying neutralizing activities pinpointed key structure-activity correlates. By using a robust in silico docking method, one promising inhibitor named golvatinib was successfully identified from the DrugBank Database. In vitro assays confirmed its ability to block viral infection and revealed its neutralizing mechanism. Our approach could be useful in the design of effective drugs for picornavirus infections.

## Introduction

Over the past 2 decades, progress in understanding human infections caused by hepatitis A virus (HAV) has been eclipsed by the priority of combating persistent hepatitis B virus (HBV) and hepatitis C virus (HCV) infections. HAV, the most important agent for enterically transmitted viral hepatitis, is distributed worldwide and infects all age groups [1]. The global burden of HAV has not abated. Approximately 1.5 million clinical cases of HAV occur annually despite the availability of an effective vaccine [2,3]. Hepatitis A as an infectious disease strongly correlates with income, hygiene, and living conditions [4]. Areas with poor hygiene and living conditions continue to be under constant threat of HAV outbreaks [4]. More recently, HAV has also started to become a new public health concern in well-developed, economically advanced countries due to the lack of natural or vaccine-induced acquired immunity to HAV in many adults [5,6]. In the past year, more than 649 people throughout California have been reported to be infected with HAV. Among these, 417 required hospitalization, and 21 patients died, making this the largest outbreak in the United States in the past 20 y [7]. Development of antiviral therapy against HAV infection is urgently needed.

HAV, transmitted via the fecal–oral route, is a positive-sense, single-stranded RNA icosahedral virus belonging to the genus *Hepatovirus* within the Picornaviridae family [8]. The 7.5 kb genome of HAV contains a single open reading frame (ORF) that encodes a giant polyprotein [9]. The polyprotein is processed by a viral protease (3C^pro^) into 3 polypeptide intermediates, namely, P1–P3 [9]. P1 is subsequently further processed into 3 structural proteins, VP0 (a precursor for VP2 and VP4), VP3, and VP1, which self-assemble into a spherical capsid with icosahedral symmetry [10]. Five copies of the VP1 capsid protein surround the icosahedral 5-fold axes. Three copies of VP2 and VP3 alternate at the 3-fold axes, and 2 copies of VP2 abut each other at the 2-fold axes [11].

Although a limited number of antigenic sites located on the HAV capsid have been revealed by escape mutants, the antigenicity of HAV is largely uncharacterized [12,13]. Our recent study involving the structure of a complex of HAV with its neutralizing monoclonal antibody (NAb), R10, extended the previously unreported VP2 antigenic sites [14]. Unlike other picornaviruses, HAV is extremely stable, both genetically and physically. So far, 6 genotypes of human HAV have been identified [15] but with only a single serotype, suggesting that HAV has highly conserved antigenic sites [16,17]. The low antigenic variation might be attributed to its highly deoptimized codon usage [18]. A systematic and comprehensive study of the antigenic characteristics of HAV and neutralizing mechanisms could facilitate the design of effective small-molecule antivirals targeting HAV.

We set out to clarify the molecular basis for the antigenicity of HAV by characterizing 4 NAbs with varying neutralizing activities against the virus. We sought this information for rationally designing antiviral inhibitors. Here, we report the characterization of 4 highly potent NAbs: F4, F6, F7, and F9. Furthermore, we have analyzed the experimentally derived high-resolution structures of HAV bound to the 4 NAbs as well as the previously reported R10-HAV structure to identify conserved epitopes for gaining key structure-activity correlates. Using a robust in silico docking method, we have screened the DrugBank Database and have identified 1 promising inhibitor named golvatinib. Cell-based antiviral assays have confirmed the ability of golvatinib to block infections caused by HAV.

## Results

### Characterization of F4, F6, F7, and F9 monoclonal antibodies

**Fig 1:**
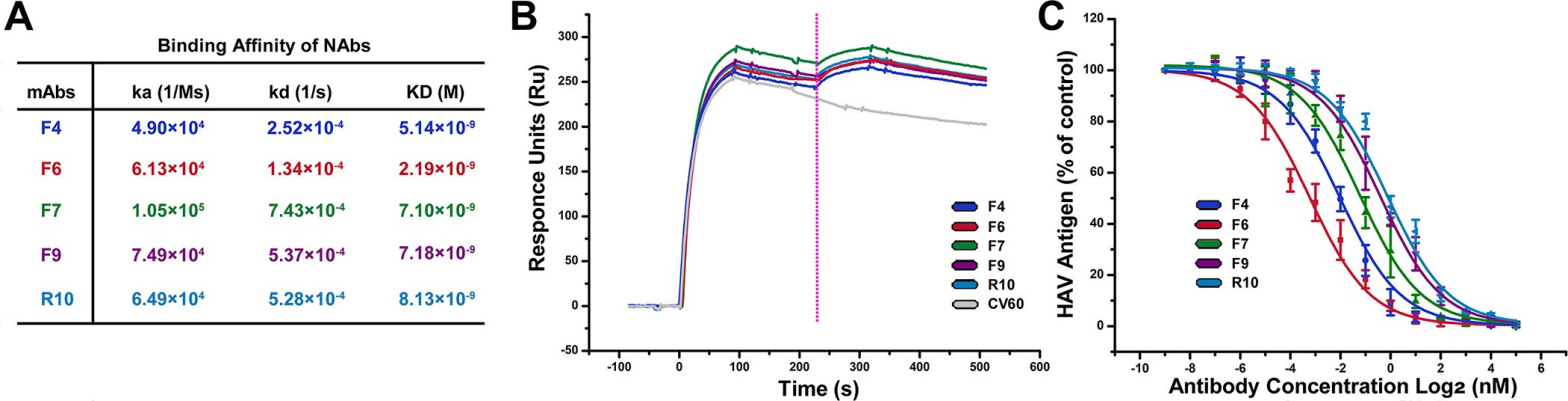
Characterization of F4, F6, F7, F9, and R10 mAbs. (A) SPR assays for characterizing binding affinities between Fab of F4, F6, F7, F9, R10, and HAV particles. The binding affinity is depicted in terms of KD (equilibrium dissociation constant, KD = kd/ka), which are listed in the figure. (B) Competition studies among the 4 different anti-HAV NAbs. The CM5 chip (BIAcore, GE Healthcare) fixed with HAV particles was first saturated with indicated R10. The capacity of an additional binding was monitored by measuring further shifts after injecting the second antibody in the presence of the first one. The pink dotted vertical line represents the second Nab’s loading time. CV60 (gray curve) was used as a non-HAV binding control. (C) Neutralization of HAV by F4, F6, F7, F9, and R10. F4 (blue curve), F6 (red curve), F7 (green curve), F9 (purple curve), and R10 (cyan curve) were used to block HAV infection at different concentrations by detecting de novo synthesized viral capsids. Data showing the levels of inhibition of virus are represented as the percentage of HAV antigen relative to antigen in the control wells. The values represent means of results from triplicate wells with SDs. The underlying data of panels B and C can be found in S1 Data.; Fab, fragment of antigen binding; HAV, hepatitis A virus; mAb, monoclonal antibody; NAb, neutralizing monoclonal antibody; SPR, surface plasmon resonance.

To shed further light on the nature of the antigenicity of HAV, 2 rounds of monoclonal antibodies (mAbs) were generated. R10, an NAb with a 50% neutralization concentration value (neut_50_) of approximately 2 nM [14], was produced in the first round, and over 30 mAbs were screened during the second round. Of these later antibodies, 4 antibodies, named F4, F6, F7, and F9, are NAbs. Surface plasmon resonance (SPR) experiments showed that the 5 NAbs bind to HAV with a high affinity in the nanomolar range (Fig 1A, S1 Fig). A number of NAbs with similar affinities are virus specific (e.g., dengue virus-specific human mAb 5J7; Japanese encephalitis virus-specific mAbs 2F2 and 2H4) and exhibit exceptionally potent neutralizing activities [19,20]. To investigate whether these 5 NAbs recognize different epitopes or the same patch of epitopes, we performed a competitive binding assay. Briefly, the CM5 chip (BIAcore, GE Healthcare), fully occupied with HAV, was initially saturated with R10, and additional binding with another NAb was evaluated. The CV60 mAb (an mAb against Coxsackievirus A16) was used as a negative control. Binding of R10 blocks the attachment of other 4 NAbs to HAV (Fig 1B), suggesting that these 5 NAbs may bind to the same patch of epitopes or at least partially overlapped epitopes. To characterize neutralizing activities, these 5 NAbs were evaluated for their abilities to prevent HAV infection. Of note, all 4 NAbs generated from the second round showed potent neutralizing activities, of which F6 exhibited the strongest neutralizing activity (a neut_50_ value of approximately 0.1 nM, which was 20-fold more potent than R10 [Fig 1C]).

### Structures of HAV in complex with its NAbs F4, F6, F7, and F9

**Fig 2:**
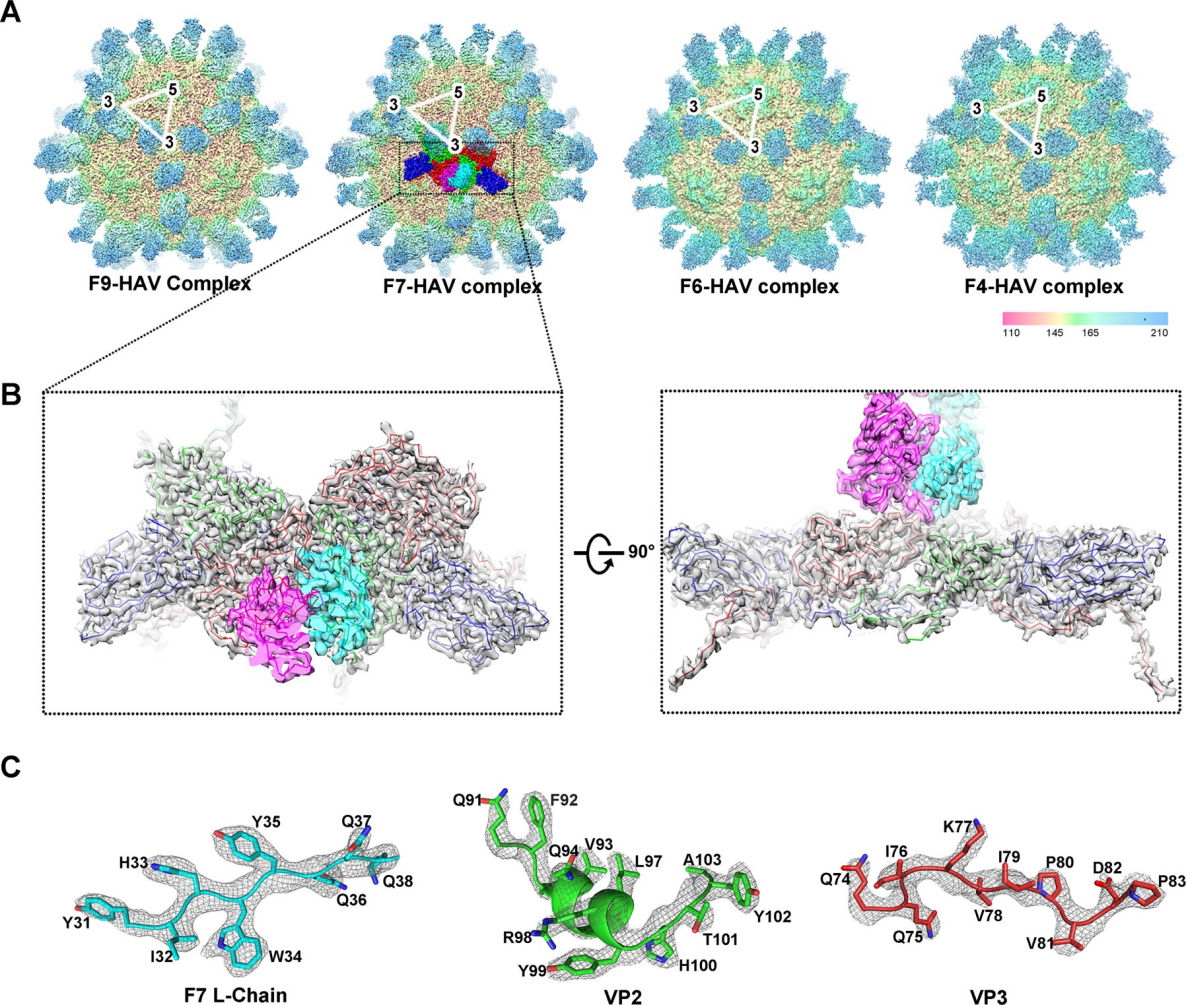
Cryo-EM structures of F4-Fab-HAV complex, F6-Fab-HAV complex, F7-Fab-HAV complex, and F9-Fab-HAV complex. (A) The maps of F4, F6, F7, and F9 Fab in complex with full particles are colored based on the radial distance (Å) of the capsid and Fab from the particle center. Only the front half of each type of particle is shown. Triangles indicate an icosahedral asymmetric unit. (B) The typical NAb’s Fab (F7) binds to the viral surface along the pentamer interface between the 2-fold and 3-fold axes. (C) The quality of Fab-HAV complex density map (gray) is illustrated by fit of backbone and side chains for 3 separate structures of F7-Fab-HAV complex. cryo-EM, cryo-electron microscopy; Fab, fragment of antigen binding; HAV, hepatitis A virus; Nab, neutralizing monoclonal antibody.

To define precisely the atomic determinants of the interactions between these 4 NAbs and HAV, structural investigations of HAV in complex with fragment of antigen binding (Fab) from its NAbs were carried out. Cryo-EM micrographs of F4-Fab-HAV, F6-Fab-HAV, F7-Fab-HAV, and F9-Fab-HAV complexes were recorded using a Titan Krios electron microscope (Thermo Fisher) equipped with a Gatan K2 detector (Gatan, Pleasanton, CA) (S2 Fig). The structures of F4-Fab-HAV, F6-Fab-HAV, F7-Fab-HAV, and F9-Fab-HAV were determined at resolutions of 3.90, 3.68, 3.05, and 3.79 Å with 4,536, 7,245, 16,743, and 3,798 particles, respectively, by single-particle techniques using the gold-standard Fourier shell correlation = 0.143 criterion [21] (Fig 2A, Table 1, S3 Fig and S4 Fig). Densities attributable to residue backbones and side chains were recognizable in maps (Fig 2A–2C). These maps were of sufficient quality to allow the atomic modelling of most of the HAV capsid proteins and NAb Fab.

**Table 1:** Cryo-EM data collection and atomic models refinement statistics.

	F4-Fab-HAV complex	F6-Fab-HAV complex	F7-Fab-HAV complex	F9-Fab-HAV complex
Data collection EM equipment	Titan Krios	Titan Krios	Titan Krios	Titan Krios
Voltage (kV)	300	300	300	300
Detector	K2	K2	K2	K2
Pixel size (Å)	1.36	1.36	1.36	1.36
Electron dose (e-Å-2)	30	30	30	30
Defocus range (μm)	1.25–3	1.25–3	1.25–3	1.25–3
**Reconstruction**				
Software	Relion 1.4	Relion 1.4	Relion 1.4	Relion 1.4
Particles selected	4,662	7,449	16,975	3,912
Number of used particles	4,536	7,245	16,743	3,798
Final resolution (Å)	3.9	3.68	3.05	3.79
**Model building**				
Software	Coot	Coot	Coot	Coot
**Refinement**				
Software	Phenix	Phenix	Phenix	Phenix
**Model statistics**				
**Ramachandran statistics**				
Favored (%)	95.52	93.25	94.76	92.60
Allowed (%)	4.37	6.59	5.13	7.41
Outliers (%)	0.11	0.16	0.11	0.05
Rotamer outliers (%)	0.00	0.00	0.00	0.00
**R.m.s.d**				
Bond lengths (Å)	0.021	0.010	0.016	0.013
Bond angles (°)	1.511	1.043	1.015	0.992

**Abbreviations:** cryo-EM, cryo-electron microscopy; Fab, fragment of antigen binding; HAV, hepatitis A virus; R.m.s.d. root-mean-square deviation.

**Fig 3:**
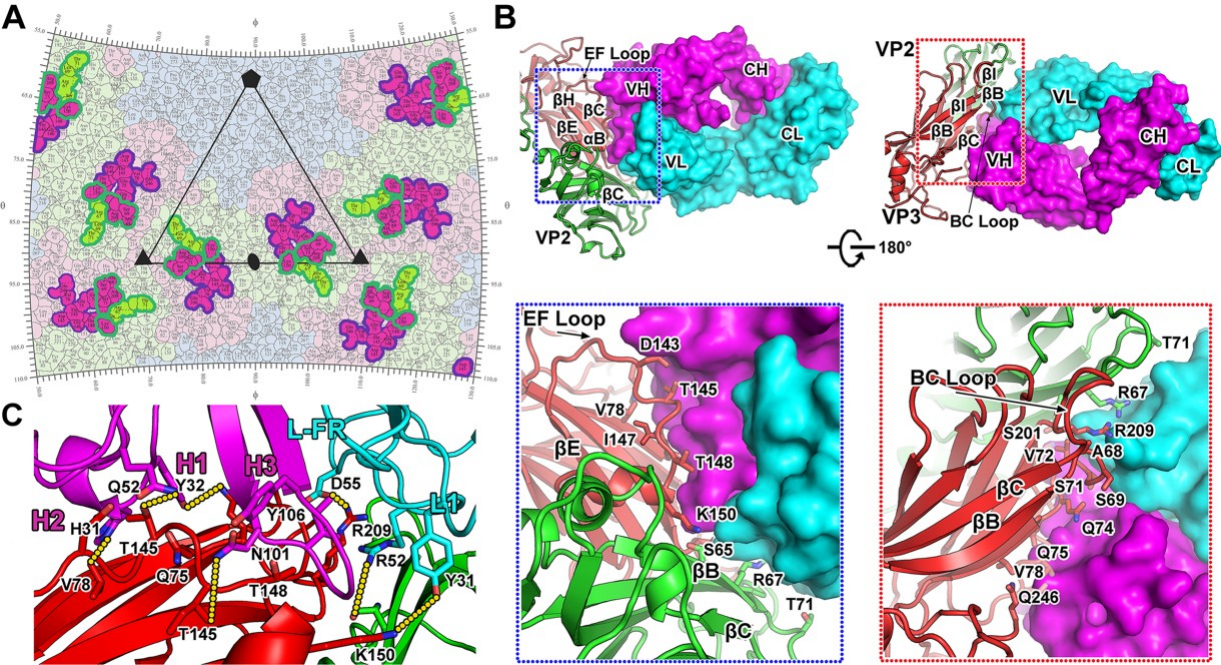
Typical interaction between HAV with F6 Fab. (A) The F6 footprints on the HAV surface. The figure shows a 2D projection of the HAV surface produced using RIVEM [25]. Residues of VP1, VP2, and VP3 are outlined in pale blue, green, and purple, respectively; residues involved in binding to F6 are shown in brighter colors corresponding to the protein chain they belong to. The footprints of F6 heavy and light chains are indicated by magenta and green lines, respectively. Five-, 3-, and 2-fold icosahedral symmetry axes are marked as for 1 icosahedral asymmetric unit. (B) The front and back view of the interaction between F6 Fab and HAV capsid proteins. F6 Fab binds to the BC loop, EF loop, C terminal of a VP3, and BC loop from another VP2. HAV capsid proteins and the Fab molecule are shown in cartoon format, with VP1, VP2, VP3, light chain, and heavy chain colored in blue, green, red, cyan, and magenta, respectively. And below, the cartoon representation of the interacting residues on capsid proteins, the left is the front view, and the right is the back view. The residues in VP2 and VP3 involved in the interactions with F6 Fab are shown as sticks. (C) Interaction between F6 Fab and HAV capsid proteins. Some residues involved in the formation of hydrogen bonds are shown as sticks and labeled. VP2, VP3, light chain, and heavy chain are colored in green, red, cyan, and magenta, respectively. Three CDRs of the heavy chain and epitope on VP2 and VP3 are highlighted by black outlines CDR, complementary determining region; Fab, fragment of antigen binding; HAV, hepatitis A virus.

The structures of these 5 complexes are almost indistinguishable. Differences are observed in the residues of the common complementary determining regions (CDRs) of NAbs (r.m.s.d. for 12,473 Ca atoms less than 1.25 Å), which are consistent with the results of the competitive binding assays (Fig 2A and S5 Fig). There are 60 copies of NAb Fabs (probably fully occupied) bound to the virus in accordance with the level of electron density for the Fab (Fig 2A). Possibly correlated with its unusual stability, HAV capsid proteins exhibit no notable conformational changes upon binding to any NAbs. Unlike EV71 or other picornaviruses in which several distinct patches for neutralizing epitopes have been reported [20,22,23,24], all 5 NAb Fabs encircle edges of the pentameric building blocks of the virus, between the 2-fold and 3-fold axes (Fig 2A and Fig 3A). Examination of the possibility of binding of 2 arms of an immunoglobulin-G (IgG) molecule to the HAV surface showed that any 2 adjacent Fabs binding to the capsid could indeed mimic the 2 arms of a single IgG molecule (S6 Fig). Therefore, the IgG avidity for all 5 NAbs might be observed due to 2 Fab arms of an IgG on the surface of HAV being sufficiently close. To explore the mechanism of neutralization, real-time reverse transcription PCR (RT-PCR) assays were performed to quantify the virus remaining on the cell surface, following exposure to antibodies’ previrus attachment to cells at 4°C. The results reveal that these NAbs prevent HAV attachment to the permissive 2BS cell surface (S7 Fig). In summary, the high potencies of all 5 NAbs could be due to several reasons, including (1) higher avidity of the bivalent form of antibody, (2) the ability of the bivalent antibody to aggregate virus particles [14], and (3) efficient block viral attachment to the host cell.

### Specific interactions between HAV and its NAbs

As expected, all Fabs exhibit a similar mode of binding, in which 1 Fab binds across the interface between the pentamers, interacting with VP2 and VP3′ from different pentamers (Fig 2). The footprints of the 5 NAbs cover interaction areas ranging from approximately 970 Å^2^ to 1,290 Å^2^, of which approximately 60% (approximately 630 Å^2^) and approximately 40% (approximately 440 Å^2^) are contributed by the heavy-chain and light-chain variable domains, respectively (Fig 3A). In line with this observation, F6 epitope contains more amino acid residues than other epitopes (S1, S2, S3, and S4 Tables), which is consistent with the results of binding affinities and neutralizing activities (Fig 1). Given the fact that the F6 exhibits the most potent antiviral activity, the epitope analysis of these 5 NAbs is representative of the F6 mAb. The heavy chain predominantly binds to the BC loop and EF loop of VP3, whereas the light chain binds to the BC loop of VP2 and the BC loop of VP3 (Fig 3B and S1 Table). The epitopes on HAV capsid include residues S65, R67, and T71 in the BC loop and A198 and S201 of VP2; A68, S69, D70, S71, V72, G73, Q74, Q75, K77, and V78 in the BC loop of VP3; and L141, D143, T145, G146, I147, T148, L149, and K150 in the EF loop of VP3 (Fig 3B and S1 Table). The region of the F6 Fab that binds HAV comprises 4 of the 6 common CDRs: H1 (residues 28–32), H2 (residues 52–57), H3 (residues 100–106), and L1 (residues 30–31) with, unusually, additional interactions contributed by the light-chain framework region (L-FR; residues 45–55; Fig 3C and S1 Table). The antibody components of these interactions include residues Y31, R45, Y48, S51, R52, L53, D55, and Q59 from the light chain and residues N28, Q30, H31, Y32, Q52, T53, N54, T56, Y57, R98, N101, I102, E103, C104, H105, and Y106 from the heavy chain (Fig 3C). Tight binding between the F6 fab and HAV capsid is facilitated by 33 hydrogen bonds and 9 salt bridges (Fig 3C).

### Structural basis of NAb activities

**Fig 4:**
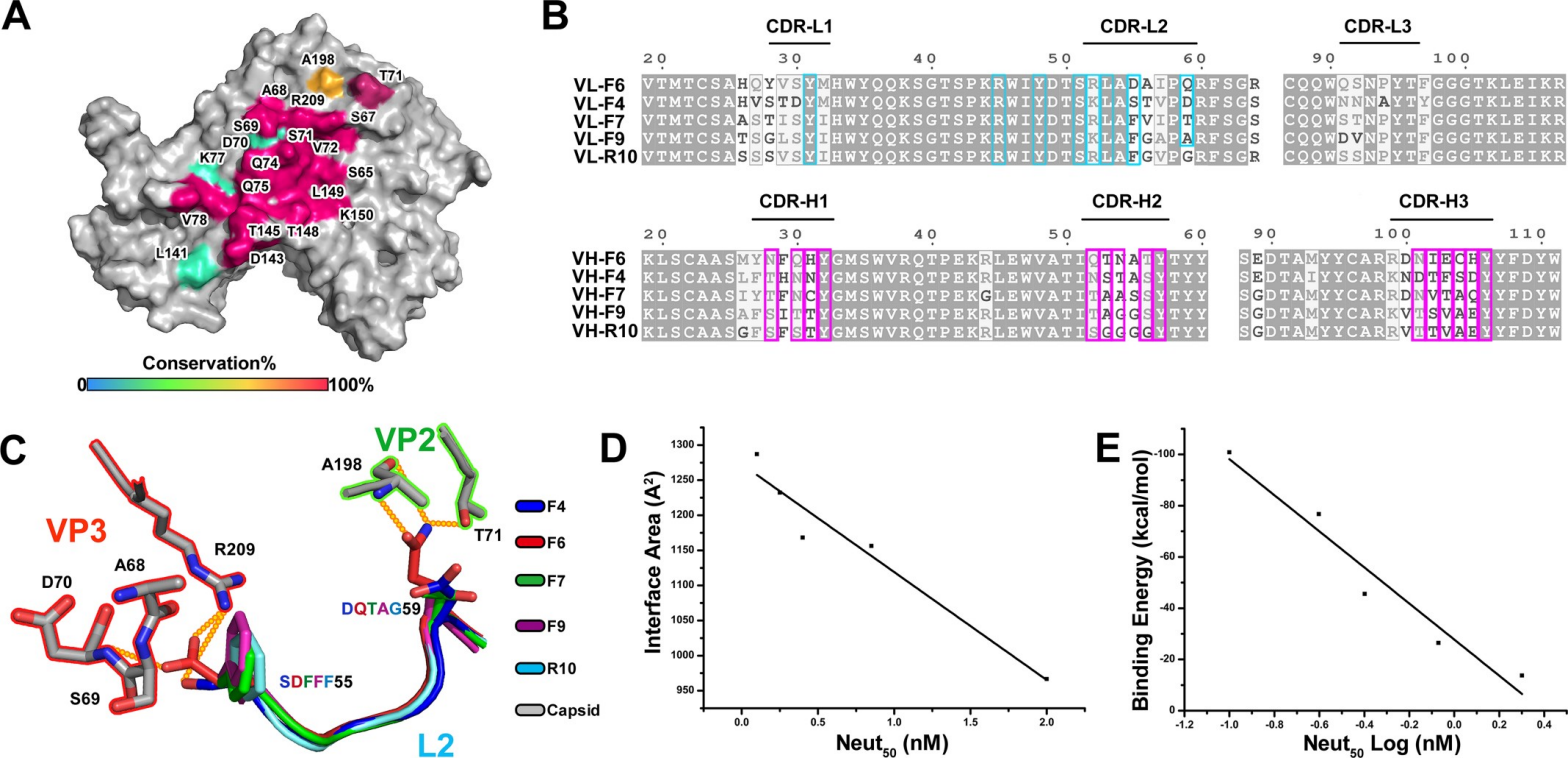
Analysis of the conservation and structure activity correlates of F4, F6, F7, F9, and R10 Fab. (A) The conservation analysis of epitopes recognized by F4, F6, F7, F9, and R10. The adjacent VP2 and VP3 of capsids are shown in surface representation with epitope residues colored according to conservation values. (B) ESPript [28] representation of sequence alignment of the variable regions of light chains and heavy chains of F4, F6, F7, F9, and R10. Residues in light chain and heavy chain that interact with HAV capsid proteins are outlined in cyan and purple, respectively. (C) Superposition of partially variable regions of light chains and heavy chains of F4, F6, F7, F9, and R10 are colored in blue, red, green, purple, and cyan. The interacted epitopes of HAV capsid are colored in gray. (D) The correlation between neutralization activity and interface area. (E) The correlation between neutralization activity (log) and binding energy. The underlying data of panels D and E can be found in S1 Data. CDR, complimentary determining region; Fab, fragment of antigen binding; HAV, hepatitus A virus.

Structures of HAV in complex with 5 NAbs reveal that epitopes on HAV locate within the same patch and are extremely conserved (Fig 4A), which is substantially different when compared to other picornaviruses, e.g., at least 4 regions of the epitopes recognized by its NAbs are mapped in EV71 [26,27]. In line with neutralizing activities, F6 epitope has 3 extra residues (D70, K77, and L141 of VP3) when compared to others, and R10 possesses the least number of epitope residues (all the NAbs recognized the A198 of VP2 except R10) (Fig 4A). These 5 NAbs share high sequence similarities at the framework region but bear relatively low sequence identities (approximately 35%) at the CDRs (Fig 4B). In spite of variations in the sequences, these 5 CDRs involved in the interactions with HAV adopt an indistinguishable configuration and a similar binding mode (Fig 2, Fig 4B, and S4 Fig). As expected, further tight binding of F6 and F4 to HAV is made possible by the additional hydrogen bonds and charge interactions formed by the antibodies (Fig 4C). Furthermore, residues in R10 that interact with HAV are also fewer in number than those observed for other NAbs. To decipher the structure activity correlates between HAV-NAb interactions and neutralizing activities, the interaction interface areas and binding energies were calculated and then compared with their neutralizing activities (S5 Table). We assembled a data set of 5 NAbs inhibition data for HAV and generated correlation plots between the Neut_50_ values and the area and energy of interaction, which produced a compelling correlation of 0.93 and 0.94, respectively (Fig 4D–4E). These analyses suggest two lessons: (1) epitopes revealed by NAbs on HAV are good targets for drug design; and (2) the more robust binding of NAbs to the epitopes, the better the antiviral activities.

### A single, conserved antigenic site

**Fig 5:**
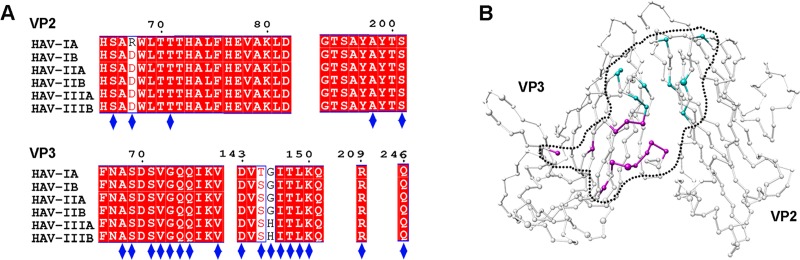
The single, conserved antigenic site on the surface of HAV. (A) Multiple-sequence alignment analysis of the 6 genotypes of human HAV capsids. The alignment results are displayed with the program ESPript [28]. The epitopes that interacted with the 5 NAbs are marked by blue diamonds. (B) Residue conservation mapped onto the HAV capsid (VP2 and VP3) using Consurf [29] (residues are displayed as spheres in variable sizes; conserved residues are shown smaller, and variable residues are shown as bigger), based on the alignment of all the virus sequences. Residues involved in interactions with heavy and light chains are shown as magenta and cyan spheres, respectively. The boundary of epitope is indicated with a black dotted circle. HAV, hepatitis A virus; NAb, neutralizing monoclonal antibody.

To date, 6 genotypes of human HAV have been identified but with only a single serotype. This indicates that these 5 NAbs are likely to bind strongly to the 6 human HAVs and could be capable of preventing human HAV infections. Sequence and structural analyses show that the residues constituting the epitopes are 87.5% identical and 94.6% conserved, with only 3 out of 21 contacting residues (residue 67 of VP2, residues 145 and 146 of VP3) being moderately conserved (50%–85%), and the remaining residues completely conserved (100%) (Fig 5A and Fig 5B). The variation rate for the epitopes is even slightly lower than that for the whole capsid (P1, approximately 94.3% conserved), highlighting a single, conserved antigenic site for HAV.

### In silico screening for identifying anti-HAV drug candidates

**Fig 6:**
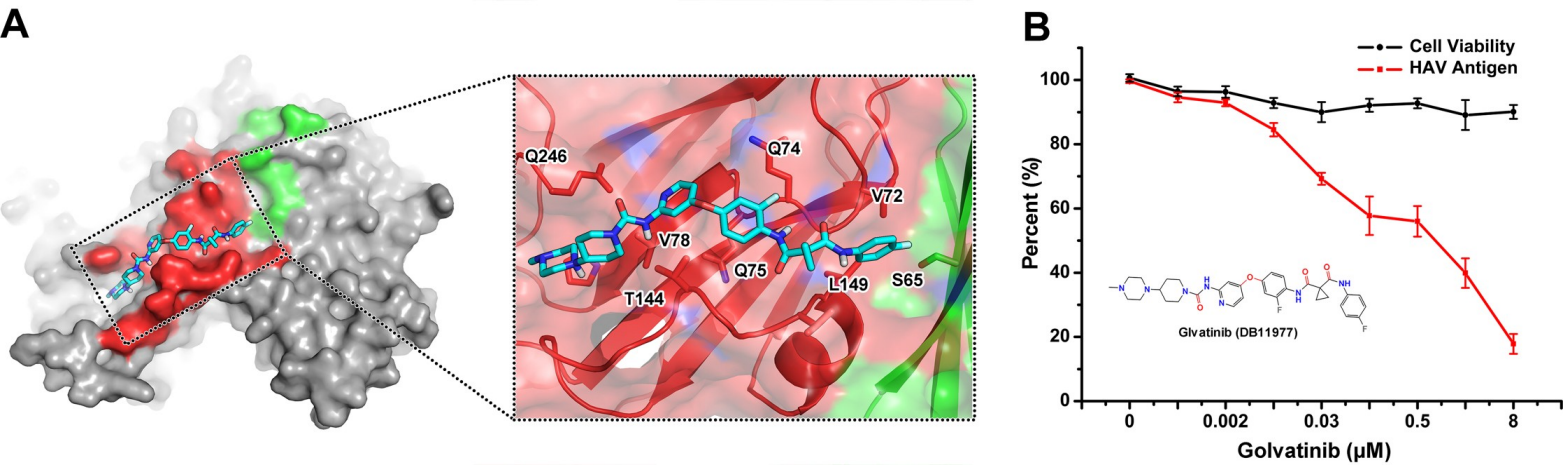
In silico–designed anti-HAV drug candidates. (A) The overall structure of golvatinib and the binding pocket as well as the interaction details between golvatinib and HAV capsid. The binding pocket is shown as a secondary structure representation and is colored in gray; the key residues are shown as a ball and stick representation. (B) The assays of cytotoxic and inhibitory effects of screened compound on 2BS cells. 2BS cells were cultured in the absence or presence of various concentrations of compounds and incubated at 34°C for 7 d. Viable cell number was determined by LDH release assay using the CCK-8 kit (Sangon Biotech, Shanghai). Data are presented as mean ± SD of 3 independent experiments. Random errors within reasonable error range (<5%) exist. Various concentrations of inhibitors were preincubated with HAV for 1 hour at room temperature before infection of 2BS cells. The inhibitory abilities of screened chemicals were evaluated by determining HAV antigen content using indirect ELISA after 7 d of infection. Values are mean ± SD. Experiments were repeated in triplicate. The underlying data of panel B can be found in S1 Data. CCK-8, cell counting kit-8; ELISA, enzyme-linked immunosorbent assay; HAV, hepatitis A virus; LDH, lactate dehydrogenase.

Given the fact that a single, conserved antigenic site exists in HAV and the key structure-activity correlates based on the antigenic site have been established, we next used the structural data to rationally design and screen potent compounds against HAV targeting the antigenic site. These residues composing the antigenic site are distributed on both sides of a long “gully,” which forms a potential inhibitor binding pocket (Fig 6A). Results of our previous study have also indicated that the “gully” area might be critical for HAV receptor binding [14]. We postulated, on the basis of inspection of the HAV-NAbs binding interface, that a tight binder (a compound) mimicking the NAbs might efficiently block HAV entry and infection.

To test this hypothesis, we scanned in silico the DrugBank database (https://www.drugbank.ca/), using Phase version 3.7 [30], Glide version 6.1 [31] to identify potential tight binders. Briefly, the 4 key residues 31, 32, 101, and 102 from the heavy chain of the 5 NAbs, which made the greatest contributions to the specific action of antigen–antibody, were selected as a reference structure for pharmacophore modeling. The generated pharmacophore was used to screen the drugs database of DrugBank. A total of 2,588 drugs were screened. Then, all selected drugs were docked to the antigenic site, and the top-ranked 4 molecules were selected. Distinct from the others, compound 3 has the best glide score (S6 Table). Therefore, compound 3, named golvatinib (DB11977), was predicted to bind to the “gully” much stronger than others (Fig 6B). As expected, in this docking pose, golvatinib contacts with the epitope residues, including S65 from VP2 and V72, G73, Q74, Q75, V78, P79, T144, T148, L149, and Q246 from VP3, via hydrophilic and hydrophobic interactions (Fig 6A). We therefore also measured the inhibitory activities of golvatinib by in vitro studies in 2BS cells. We used 100 50% tissue culture infective dose (TCID_50_) virus in the presence of different concentrations of the compounds and exposed control wells to the equivalent concentration of solvent (DMSO) to ensure no effects on uninfected cells or on virus titer (S8 Fig). The compound golvatinib exhibited a potent antiviral activity, with a 50% inhibitory concentration (IC_50_) of approximately 1 μM, inhibiting the viral titer to below 15% at concentrations over 8 μM (Fig 6B). Meanwhile, no notable cytotoxic effect of golvatinib at concentrations of 0.0005 to8 μM was observed (Fig 6B). The measured antiviral activity of golvatinib is in agreement with that predicted in silico. As expected, golvatinib, like the 5 NAbs, inhibits HAV infection by blocking attachment to the host cell (S9 Fig). Due to the partial overlapped binding sites of the NAbs and of golvatinib (Fig 6A), it is quite possible that they are capable of competing each other to attach the HAV surface. In addition, the binding of golvatinib to the HAV does not alter its particle stability (S9 Fig), which is consistent with our previous results that stabilization or destabilization is unlikely to be the major neutralization mechanism in our study systems [14].

## Discussion

Attachment of the virus to its cellular receptors located on the surface of the host cell and uncoating of the virus leading to the release of the viral genome into host cells are regarded as the 2 key steps for the successful entry of nonenveloped viruses, including picornaviruses, into host cells [32]. Neutralizing antibodies block the entry of viruses into host cells by blocking the attachment of the virus to the cellular receptor [33], overstabilizing the virus [34], preventing the release of viral genome [27], or physically destabilizing the capsid of the virus [22]. In our previous study, we demonstrated that R10, a HAV-specific neutralizing antibody, neutralizes HAV infection by preventing the binding of the virus to its putative receptor T-cell immunoglobulin and mucin-containing domain 1 (TIM-1) [14]. Recent evidences suggests that TIM-1 is not an essential receptor for the naked (unenveloped) HAV but rather an attachment factor for quasi-enveloped virions [35], making the bona fide receptor(s) elusive. Therefore, it is challenging to verify whether the binding of NAbs or golvatinib blocks the interactions between HAV and its bona fide receptor. Many picornaviruses use cell-surface molecules belonging to the immunoglobulin superfamily (IgSF) as their cellular receptors, which usually consist of tandem repeats of between 1 and 5 Ig-like domains to interact with viruses [36]. Given the fact that R10 competitively blocked TIM-1 Ig V binding to HAV [14], it is possible that the bona fide receptor might be from the IgSF. In this study, the antigen binding site of the newly screened NAbs (F4, F6, F7, and F9) maps to the same epitope on the surface of HAV as that identified for R10, suggesting that the binding sites of the 4 NAbs and the bona fide receptor may overlap.

Previous studies have shown that residues S102, V171, A176, and K221 of VP1 and D70, S71, Q74, and 102–121 of VP3 are part of the neutralizing epitopes [13]. However, structural analysis reveals that these putative epitope residues are forming 2 clusters that are separated by a distance of 40 to 50 Å on the HAV surface, suggesting that these residues are unlikely to form a single antigenic site. The high-resolution structures of HAV in complex with 4 NAbs described in this study coupled with the results of our previous studies on HAV-antibody complexes [14] further verify the fact that VP2 (but not VP1), as well as VP3, form a single, conserved antigenic site on the surface of HAV, which differs radically from the architecture of the antigenic sites of other picornaviruses [37,38]. However, our studies cannot exclude the possibility of the likely existence of a second neutralizing antigenic site involving residues in VP1, yet ill-defined on the viral capsid. Additionally, the neutralizing epitopes should differ with those of binding but non-neutralizing antibodies, which needs to be further investigated. The single, conserved antigenic site we identify could serve as an excellent target for structure-based drug design.

Although hepatitis A is a vaccine-preventable disease [39], an anti-HAV drug would be indispensable for treating fulminating infections. In this study, about 2,588 drug candidates (compounds) from the DrugBank database were selected for in silico docking studies. One of the candidates predicted to interact with the conserved antigenic site by the docking studies exhibited excellent antiviral activity without any notable cytotoxicity. Therefore, based on the preclinical evaluation of its cytotoxicity and pharmacodynamics, golvatinib, previously investigated for the treatment of platinum-resistant squamous cell carcinoma of the head and neck [40], could act as a lead compound for anti-HAV drug development.

In summary, we have used a combined experimental and computational approach starting from a number of NAbs targeting a single, conserved antigenic site located on the surface of a complete viral capsid to obtain, in a single round of design, a potent micromolar-range drug candidate that is effective and safe and has many drug-like properties.

## Materials and methods

### Ethics statement

Animals were bred and maintained under specific pathogen-free (SPF) conditions in the institutional animal facility of the Institute of Biophysics, Chinese Academy of Sciences. All animal experiments were performed with protocols (protocol numbers VET102, VET201, VET203, and VET301) approved by the Animal Care and Use Committee of Institute of Biophysics, Chinese Academy of Sciences.

### Particle production and purification

HAV virus genotype TZ84 (HAV IA genotype) was used to infect 2BS cells at a multiplicity of infection (MOI) of 0.2 at 34°C. Particle production and purification have been described previously [11].

### Production of Fab fragments

F4, F6, F7, and F9 were purified from mouse ascites with a protein A affinity column (GE). The Fab fragment was generated using a Pierce FAB preparation Kit (Thermo Scientific), according to the manufacturer’s instructions. Briefly, after removal of the salt using a desalting column, the antibody was mixed with papain and then digested at 37°C for 6 h. The Fab was separated from the Fc fragment by using a protein A affinity column. Then, Fab was loaded onto a Hitrap Q FT column (GE). Fractions corresponding to the major peak were collected and concentrated for cryo-EM analysis.

### Binding affinity assay

The binding affinities of the 5 NAb assays were determined by SPR. These experiments were performed using a BIAcore 3,000 machine (BIAcore, GE Healthcare) in the buffer solution containing 10 mM HEPES (pH 7.4), 150 mM NaCl, and 0.005% v/v Tween 20 at 25°C. The purified HAV full particles were directly immobilized onto CM5 sensor chips (BIAcore, GE Healthcare) at concentrations equivalent to approximately 950 response units (0.3 mg/ml). Subsequently, gradient concentrations (0.0315, 0.0625, 0.125, and 0.25 μM) of purified Fab fragments of F4, F6, F7, F9, and R10 were used to flow over the chip surface. To regenerate the chip, 100mM NaOH was used. The binding affinities were analyzed using steady state affinity with the software BIAevaluation version 4.1.

### Binding competition assay

Binding competition between HAV antibodies was determined using SPR (BIAcore 3,000, GE). The entire experiment was performed at 25°C in the buffer solution containing 10 mM HEPES (pH 7.4), 150 mM NaCl, and 0.005% v/v Tween 20. The CM5 biosensor chip (BIAcore, GE Healthcare) immobilized with HAV full particles (0.3 mg/ml) was first saturated with R10 for 5 min. Afterward, the other NAbs were injected in the presence of R10 for another 3 min. CV60, an irrelevant antibody, was used as a negative control. Except for R10, all other NAbs were evaluated at a concentration of 300 nM for saturation. R10 was applied at a concentration of 900 nM. The chip was regenerated with 100mM NaOH (GE Healthcare).

### Neutralization assay

For the neutralization assay, purified mAbs at a concentration of 0.2 mg/ml were initially diluted 8-fold as stocks and then serially diluted 2-fold with DMEM containing 2% FBS; 100 μl of 2-fold antibody dilutions were mixed with 100 μl of HAV containing 100 TCID_50_ for 1 h at 37°C and then added to monolayers of 2BS cells in cell culture flasks (T25 CM^2^). Meanwhile, maintaining medium was provided as well. Each dilution was replicated 3 times along with one control that contained no antibody dilution. After 21 d of growth at 34°C, the medium was removed, and the cells were washed three times using PBS buffer; 1 ml of Trypsin/EDTA was added, and the flask was left for 3 min at 37°C. The suspended cells were freeze-thawed 5 times to collect the virus. Enzyme-linked immunosorbent assay (ELISA) was used to measure HAV antigen content. The percent inhibition was determined relative to the mean OD_450_ values of the control wells in which the virus has been incubated with medium alone.

### Cryo-EM and data collection

Purified F4, F6, F7, and F9 Fab fragments were incubated with purified HAV (at a concentration of 2 mg/ml) separately on ice for 10 min at a ratio of 120 Fab molecules per virion. A 3-μl aliquot of the mixtures of F4-Fab-HAV, F6-Fab-HAV, F7-Fab-HAV, and F9-Fb-HAV were transferred onto a freshly glow-discharged 400-mesh holey carbon-coated copper grid (C-flat, CF-2/1-2C; Protochips). Grids were blotted for 3.5 s in 100% relative humidity for plunge-freezing (Vitrobot; FEI) in liquid ethane. Cryo-EM data sets were collected at 300 kV using a Titan Krios microscope equipped (Thermo Fisher) with a K2 detector (Gatan, Pleasanton, CA). Movies (25 frames, each 0.2 s, total dose 30 e^−^ Å^−2^) were recorded with a defocus of between 1 and 2.5 μm using SerialEM [41], which yields a final pixel size of 1.35 Å.

### Image processing, 3D reconstruction, model building, and refinement

The frames from each movie were aligned and averaged for the correction of beam-induced drift using MOTIONCORR [42]. Particles from micrographs were picked automatically using ETHAN [43] and then manually screened using the boxer program in EMAN [44]. The CTF parameters for each micrograph were estimated by using a GPU accelerated program Gctf [45]. Cryo-EM structures were determined with Relion 1.4 [46] with the application of icosahedral symmetry. The initial model was created by EMAN2 [47]. A total of 4,536, 7,245, 16,743, and 3,798 particles of F4-Fab-HAV, F6-Fab-HAV, F7-Fab-HAV, and F9-Fab-HAV were used to determine structures at resolutions of 3.9, 3.68, 3.05, and 3.79 Å, respectively, as evaluated by the so-called gold standard FSC procedure between 2 half maps (threshold = 0.143) [21]. The crystal structure of HAV full particle (PDB ID code 4QPI) was used to fit the complex EM maps, and the atomic models of F4-, F6-, F7-, and F9-Fabs were built de novo into densities with the structure of R10 (PDB ID code 5WTG) as a guide, using COOT [48]. All models were further refined by positional and B-factor refinement in real space using Phenix [49] and rebuilding in COOT [48] iteratively. The final models were evaluated by Molprobity [50] functions integrated in Phenix. Data and refinement statistics are summarized in Table 1.

### Binding energy calculation

In antigen binding systems, the chain C, D, E, and G of F4 Fab-HAV, F6 Fab-HAV, F7 Fab-HAV, and F9 Fab-HAV structures were fetched out to perform MD simulation and binding energy calculations. In the 4 chains, the chain D and E were set to ligand and the chain C and G were set to receptor. The complex was solvated to TIP3P waters, and 0.1 M NaCl was added to systems as salt with soft tleap in AmberTools 16 [51].

Amber 16 was used to perform MD simulation. All 4 systems were first relaxed by 5,000-step minimization (2,000 steps, steepest descent minimizations; 3,000 steps, conjugate gradient minimization). After minimization, the system was gradually heated from 0 K to 300 K in the canonical NVT ensemble with a Langevin thermostat using a collision frequency of 2.0 ps^−1^. Initial velocities were assigned from a Maxwellian distribution at the starting temperature. Then 100 ps of density equilibration with weak restraints on the complex was followed by 500 ps of constant pressure equilibration at 300 K and 1 atm. Finally, 10 ns MD simulations for each system was conducted with the target temperature at 300 K and the target pressure at 1.0 atm. In 4 systems, Na^+^ was selected as the counter ions; the concentration of NaCl was set to 0.1 M, and ions’ parameters of Joung and Cheatham [52] were used. Electrostatics was handled using the particle mesh Ewald (PME) algorithm [53] with a 10.0 Å direct−space nonbonded cutoff. All bonds involving hydrogen atoms were constrained using the SHAKE algorithm [54], using a time step of 2.0 fs. The coordinates’ trajectories were saved every 2 ps during the whole MD runs.

MM-GBSA [55] was used to calculate the binding energy of F4, F6, F7, F9, and R10 Fab (chain D and chain E) and HAV VP2 and VP3 (chain C and chain G). In each system, 100 snapshots of the last 6 ns MD simulation were fetched out to calculate the binding energy. The entropy contributions were neglected because the same receptor was used and because the normal mode analysis calculations are computationally expensive and subject to a large margin of error that introduces significant uncertainty in the result.

The free energy for each species (ligand, receptor, and complex) is decomposed into a gas-phase MM energy, polar, and nonpolar solvation terms, as well as an entropy term, as shown in the following equation:

$$\begin{array}{l} \;\Delta G=\Delta E_{MM}+{\Delta G}_{solv}-T∙\Delta S\\ ={\Delta E}_{bat}+{\Delta E}_{vdw}+{\Delta E}_{coul}+{\Delta G}_{solv.p}+{\Delta G}_{solv.np}-T∙\Delta S. \end{array}$$

EMM is composed of Ebat (the sum of bond, angle, and torsion terms in the force field), a van der Waals term, EvdW, and a Coulombic term, Ecoul. Gsolvp is the polar contribution to the solvation free energy, often computed via the Generalized-Born (GB) approximation. Gsolvnp is the nonpolar solvation free energy, usually computed as a linear function of the solvent-accessible surface area (SASA).

### Molecular docking

The Phase program of the Schrodinger Suite 2013 [30] was used for the pharmacophore modeling. Four key residues at positions of 31, 32, 101, and 102 from the heavy chain of the 5 NAbs were selected as a reference structure for modeling. Pharmacophore sites were generated using the default set of chemical features: hydrogen bond acceptor (A), hydrogen bond donor (D), hydrophobe (H), negative ionizable (N), positive ionizable (P), and aromatic ring (R). The size of the pharmacophore box was set to 1 Å to optimize the number of final common pharmacophore hypotheses. The generated pharmacophore was used to screen the drugs database of Drugbank. The distance matching tolerance was set to 2.0 Å. A total of 2,588 drugs were screened out using this procedure.

The docking algorithm Glide [31], which is based on descriptor matching, was used to perform virtual screening and learn the interactions between small molecules and the protein structure. The structure of HAV epitopes was prepared and then used to build the energy grid. For Glide docking, the docking box was centered on the position of mass center of the 4 selected residues, and its outer box size was set to 40 × 40 × 40 Å. The scaling factor for protein van der Waals radii was set to 1.0. All 2,588 drugs were docked to the antigen binding site on HAV capsids, and the 4 molecules were selected. The compound 3, which has the best glide score, was selected finally.

### Inhibition assay of HAV infection

Approximately 2 × 10^5^ 2BS cells were seeded into each well of a 24-well plate and incubated overnight in a CO_2_ incubator supplemented with 5% CO_2_. Before virus infection, HAV (100 TCID_50_) was incubated with serially diluted concentrations (0, 0.008, 0.032, 0.125, 0.5, 2, and 8 μM) of golvatinib (MedChemExpress) for 1 h at room temperature with gentle rocking and then transferred to the plate containing 2BS cells. After adsorption for 1 h, the inoculum was removed, and the cells were supplied with fresh maintenance medium and incubated at 34°C. At 7 d post infection, the cells were lysed for ELISA to determine HAV antigen content.

### Cell viability assay

Approximately 2 × 10^5^ 2BS cells were seeded into 24-well plates and incubated overnight in a CO_2_ incubator. Inhibitors were serially diluted concentrations (0, 0.008, 0.032, 0.125, 0.5, 2, and 8 μM) and then transferred to the plate containing 2BS cells. Seven days after addition of drug, CCK-8 kit (Sangon Biotech) was used according to the manufacturer’s protocol. In brief, each well of the plate had10 μl CCK-8 solution added and was incubated 2 h at 37°C. Absorbance at 450 nm was measured by SynergyH1 microplate reader (BioTek).

### Thermofluor assay

An MX3005p RT-PCR instrument (Agilent) was used for the thermofluor assays. SYTO9 (Invitrogen) was used as a fluorescent probe to detect the presence of single-stranded RNA [56,57]. A 50 μL reaction solution was set up in the PCR plate (Agilent), containing 1.0 μg of virus plus serially diluted concentrations of golvatinib (0, 0.512, 5.12, and 51.2 μM) and 5 μM SYTO9 in PBS buffer solutions, and ramped from 25°C to 99°C with fluorescence recorded in triplicate at 1°C intervals. The RNA release (Tr) temperature was taken as the minimum of the negative first derivative of the RNA exposure.

### Viral attachment assay

HAV (100 TCID_50_) was mixed with serially diluted concentrations of golvatinib or NAbs before the virus attached to cells (2 × 10^5^) and then added to 2BS cells and incubated at 4°C for 1 h. The cells were washed three times and total cellular RNA purified using RNeasy mini kit (Qiagen), as described in the manufacturer’s instructions. Real-time quantitative PCR (qPCR) was performed using One Step SYBR PrimeScript RT-PCR Kit (TaKaRa) in a MX3005p RT-PCR instrument (Agilent). The 20-μL reaction contained 12.5 μL 2 × One Step SYBR RT-PCR Buffer III, 0.5 μL TaKaRa Ex Taq HS, 0.5 μL PrimeScript RT Enzyme Mix II, 0.5 μL each of 10 μM forward (5′-TGG AAT CAC ATT AAA GCA AGC AA-3′) and reverse (5′-GGA ACA CGA AAT CTC AAA GTT GAC T-3′) primers, 2 μL total RNA, and 4 μL RNase-free H_2_O. The thermal profile for qPCR was 42°C for 5 min for reverse transcription, 95°C for 10 s for reverse transcription inactivation; this was followed by 40 cycles of denaturation at 95°C for 10 s and annealing and extension at 60°C for 30 s. GAPDH was used as the housekeeping gene to normalize samples (forward 5′-CTG TTG CTG TAG CCA AAT TCGT-3′, reverse 5′-ACC CAC TCC TCC ACC TTT GAC-3′). The analysis of relative levels of HAV RNA in different samples was performed by comparative 2^−ΔΔCT^ method [58].

### Data deposition

The cryo-EM maps of the F4-Fab-HAV, F6-Fab-HAV, F7-Fab-HAV, and F9-Fab-HAV complexes were deposited in the Electron Microscopy Data Bank with accession number EMD-9827, EMD-9828, EMD-9829, and EMD-9830, respectively. The atomic coordinates for F4-Fab-HAV, F6-Fab-HAV, F7-Fab-HAV, and F9-Fab-HAV complexes were deposited in the PDB with accession numbers: 6JHQ, 6JHR, 6JHS, and 6JHT, respectively.

## Supporting information

S1 Fig The binding affinities of HAV capsids and F4, F6, F7, F9, and R10 Fab estimated by SPR. HAV capsids were directly immobilized onto CM5 sensor chips (BIAcore, GE Healthcare) at approximately 950 response units. Gradient concentrations (0.012, 0.037, 0.11, 0.33 μM) of purified Fab fragments of F4, F6, F7, F9, and R10 were used to flow over the chip surface. The underlying data of this figure can be found in S1 Data. Fab, fragment of antigen binding; HAV, hepatitis A virus; SPR, surface plasmon resonance.
(TIF)

S2 Fig Purification of HAV, F4, F6, F7, F9, and R10 Fab. (A) Purification of F4, F6, F7, F9, and R10 Fab. Purity of samples was assessed by SDS-PAGE analysis. (B) Zonal ultracentrifugation of a 15% to 45% (w/v) sucrose density gradient for the purification of HAV as described in the Materials and methods section. Two predominant particle types were separated; the empty particles located at approximately 27% sucrose, the full at approximately 32% sucrose. (C) SDS-PAGE analysis for determining composition of viral proteins. The dashed black line indicates that this panel is a composite image of two discontinuous lanes from the same gel. Fab, fragment of antigen binding; HAV, hepatitis A virus.
(TIF)

S3 Fig Cryo-EM images and resolution of cryo-EM maps. (A) Cryo-EM images of HAV particles complexed with F4, F6, F7, and F9 Fab. (B) The gold-standard FSC curves of complexes of F4 Fab-HAV, F6 Fab-HAV, F7 Fab-HAV, and F9 Fab-HAV. (C) Local resolution assessment. Local-resolution F4 Fab-HAV, F6 Fab-HAV, F7 Fab-HAV, and F9 Fab-HAV maps of density slices, rendered using ResMap [59], are shown. The red to blue color scheme corresponds to regions of relative low to high resolution. The underlying data of panel B can be found in S1 Data. cryo-EM, cryo-electron microscopy; Fab, fragment of antigen binding; FSC, fourier shell correlation; HAV, hepatitis A virus.
(TIF)

S4 Fig Close-up of F4, F6, F7, F9, and R10 Fab binding to HAV capsids. Closeup view of the interaction interface involving 4 of 6 CDRs on the Fab and surface capsids. F4 Fab-HAV, F6 Fab-HAV, F7 Fab-HAV, and F9 Fab-HAV are colored in blue, red, green, and purple in (A), (B), (C), and (D), respectively. CDR, complementary determining region; Fab, fragment of antigen binding; HAV, hepatitis A virus.
(TIF)

S5 Fig Superposition of the structures of F4 Fab-HAV, F6 Fab-HAV, F7 Fab-HAV, F9 Fab-HAV, and R10 Fab-HAV complexes. Structural comparisons of the 5 complexes—F4 Fab-HAV, F6 Fab-HAV, F7 Fab-HAV, F9 Fab-HAV, and R10 Fab-HAV—were superposed by pymol, and an asymmetry unit of Fab-HAV complex was colored by r.m.s.d. Fab, fragment of antigen binding; HAV, hepatitis A virus; r.m.s.d., root-mean-square deviation.
(TIF)

S6 Fig The distances between 2 adjacent Fabs. The distances between 2 adjacent Fabs are measured and labeled. Given the fact that the distance between 2 Fabs is approximately 60 Å, making low resolution surface from coordinates is done by Multi-scale Models in Chimera [60]. Fab, fragment of antigen binding.
(TIF)

S7 Fig F4, F6, F7, and F9 neutralize HAV by inhibiting attachment of HAV. The amount of virions on the cell surface was detected by RT-PCR after binding to F4, F6, F7, or F9 before the virus was allowed to attach to 2BS cells. High concentrations of NAbs prevented attachment of HAV to the cell surface when HAV was exposed to antibodies before cell attachment. Data are presented as mean ± SD of 3 independent experiments. The underlying data of panels A to D can be found in S1 Data. HAV, hepatitis A virus; Fab, fragment of antigen binding; NAb, neutralizing monoclonal antibody; RT-PCR, reverse transcription PCR.
(TIF)

S8 Fig The effects of DMSO on virus titer and uninfected cells. Various concentrations of DMSO were preincubated with HAV for 1 h at room temperature before infection of 2BS cells. The effects on virus titer were evaluated by determining HAV antigen content using indirect ELISA after 7 d of incubation. Values are mean ± SD. Experiments were repeated in triplicate. The assays of cytotoxic was determined by LDH release assay using the CCK-8 kit (Sangon Biotech, Shanghai) after 7 d of incubation. Data are presented as mean ± SD of 3 independent experiments. The underlying data of this figure can be found in S1 Data. CCK-8, cell counting kit-8; ELISA, enzyme-linked immunosorbent assay; HAV, hepatitis A virus; LDH, lactate dehydrogenase.
(TIF)

S9 Fig Golvatinib inhibits HAV infection by blocking attachment to the host cell without altering its particle stability. (A) Amount of virions on the cell surface was detected by RT-PCR after bind to golvatinib before the virus was allowed to attach to cells. Data are presented as mean ± SD of 3 independent experiments. (B) The stabilities of HAV particles in the presence of a serially diluted concentrations of golvatinib (0, 0.512, 5.12, and 51.2 μM) were determined by thermofluor assay using the dye SYTO9 to detect RNA exposure [57]. The binding of golvatinib to the HAV does not alter its particle stability, even in a highly concentrations of golvatinib. The underlying data of panels A and B can be found in S1 Data. HAV, hepatitis A virus; RT-PCR, reverse transcription PCR.
(TIF)

S1 Table Interacting residues between F4 Fab and HAV in an asymmetric unit. Fab, fragment of antigen binding; HAV, hepatitis A virus.
(DOCX)

S2 Table Interacting residues between F6 Fab and HAV in an asymmetric unit. Fab, fragment of antigen binding; HAV, hepatitis A virus.
(DOCX)

S3 Table Interacting residues between F7 Fab and HAV in an asymmetric unit. Fab, fragment of antigen binding; HAV, hepatitis A virus.
(DOCX)

S4 Table Interacting residues between F9 Fab and HAV in an asymmetric unit. Fab, fragment of antigen binding; HAV, hepatitis A virus.
(DOCX)

S5 Table Results of 5 NAbs’ binding energy, interface area calculation, and neutralizing activity. NAb, neutralizing monoclonal antibody.
(DOCX)

S6 Table The structure and glide score of selected compounds. (DOCX)

S1 Data Underlying data for the following Figs 1B, 1C, 4D, 4E, 6B, S1, S3B, S7A, S7B, S7C, S7D, S8, S9A, and S9B. (XLSX)

## Abbreviations

CDR: complementary determining region
cryo-EM: cryo-electron microscopy
ELISA: enzyme-linked immunosorbent assay
Fab: fragment of antigen binding
GB: Generalized-Born
HAV: hepatitis A virus
HBV: hepatitis B virus
HCV: hepatitis C virus
IC_50_: 50% inhibitory concentration
IgG: immunoglobulin-G
IgSF: immunoglobulin superfamily
LDH: lactate dehydrogenase
L-FR: light chain framework region
mAb: monoclonal antibody
MOI: multiplicity of infection
NAb: neutralizing monoclonal antibody
neut_50_: 50% neutralization concentration
ORF: open reading frame
qPCR: quantitative PCR
r.m.s.d.: root-mean-square deviation
RT-PCR: reverse transcription PCR
SASA: solvent-accessible surface area
SPF: specific pathogen-free
SPR: surface plasmon resonance
TCID_50_: 50% tissue culture infective dose
TIM-1: T-cell immunoglobulin and mucin-containing domain 1
