# Supplementary figures and images for: Structural basis for neutralization of hepatitis A virus informs a rational design of highly potent inhibitors
Source: PLoS Biol. 2019 Apr 30;17(4):e3000229. doi: 10.1371/journal.pbio.3000229 (PMC6493668; doi:10.1371/journal.pbio.3000229)

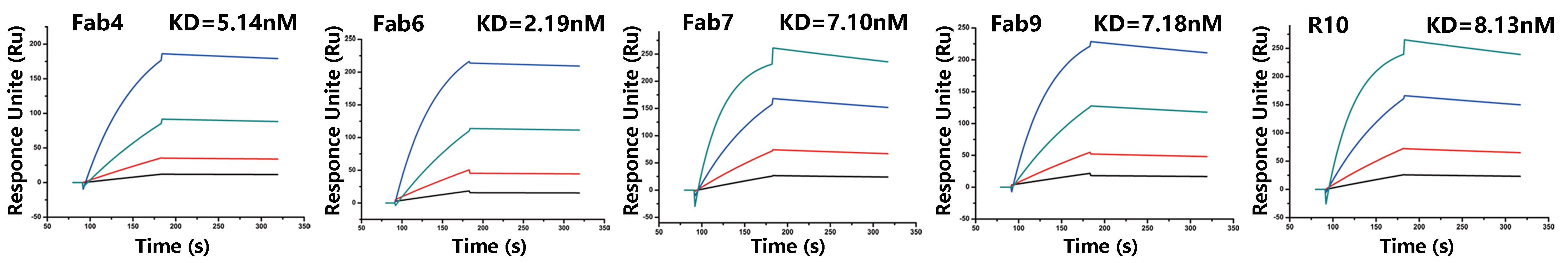

Supplement: S1 Fig — HAV capsids were directly immobilized onto CM5 sensor chips (BIAcore, GE Healthcare) at approximately 950 response units. Gradient concentrations (0.012, 0.037, 0.11, 0.33 μM) of purified Fab fragments of F4, F6, F7, F9, and R10 were used to flow over the chip surface. The underlying data of this figure can be found in S1 Data. Fab, fragment of antigen binding; HAV, hepatitis A virus; SPR, surface plasmon resonance. (TIF) [file pbio.3000229.s001.tif]

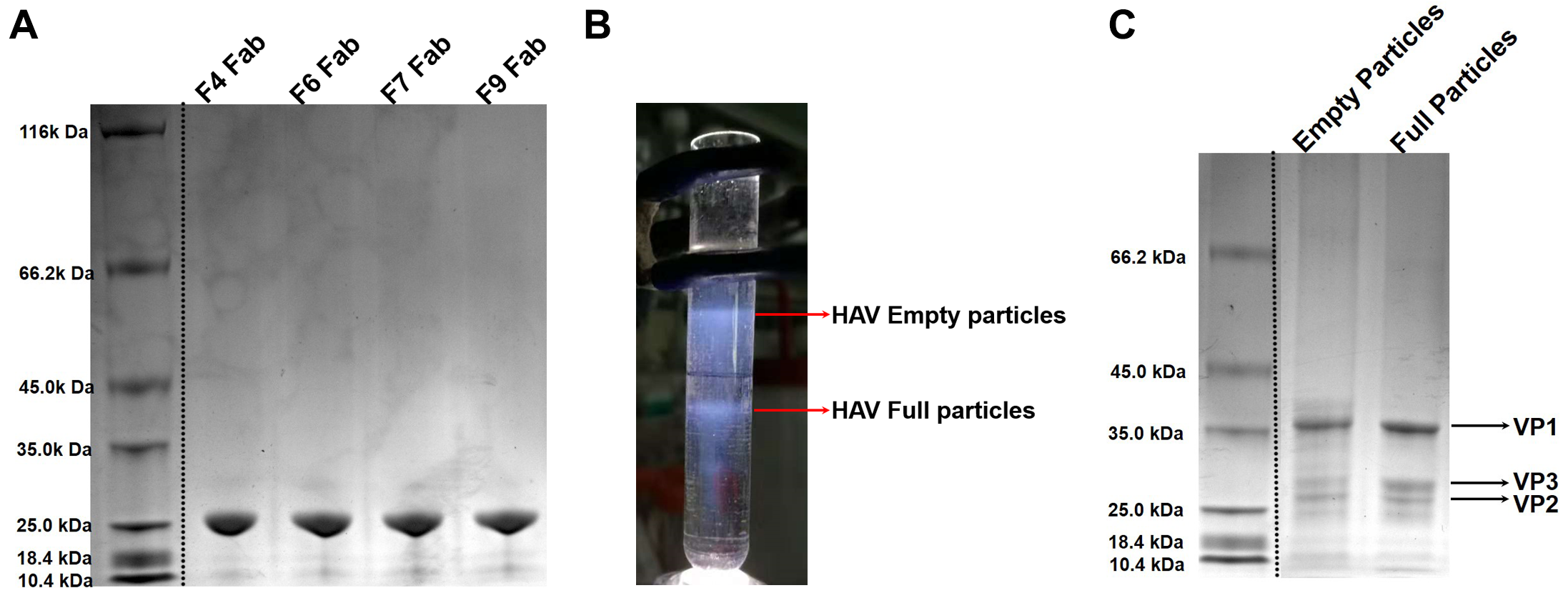

Supplement: S2 Fig — (A) Purification of F4, F6, F7, F9, and R10 Fab. Purity of samples was assessed by SDS-PAGE analysis. (B) Zonal ultracentrifugation of a 15% to 45% (w/v) sucrose density gradient for the purification of HAV as described in the Materials and methods section. Two predominant particle types were separated; the empty particles located at approximately 27% sucrose, the full at approximately 32% sucrose. (C) SDS-PAGE analysis for determining composition of viral proteins. The dashed black line indicates that this panel is a composite image of two discontinuous lanes from the same gel. Fab, fragment of antigen binding; HAV, hepatitis A virus. (TIF) [file pbio.3000229.s002.tif]

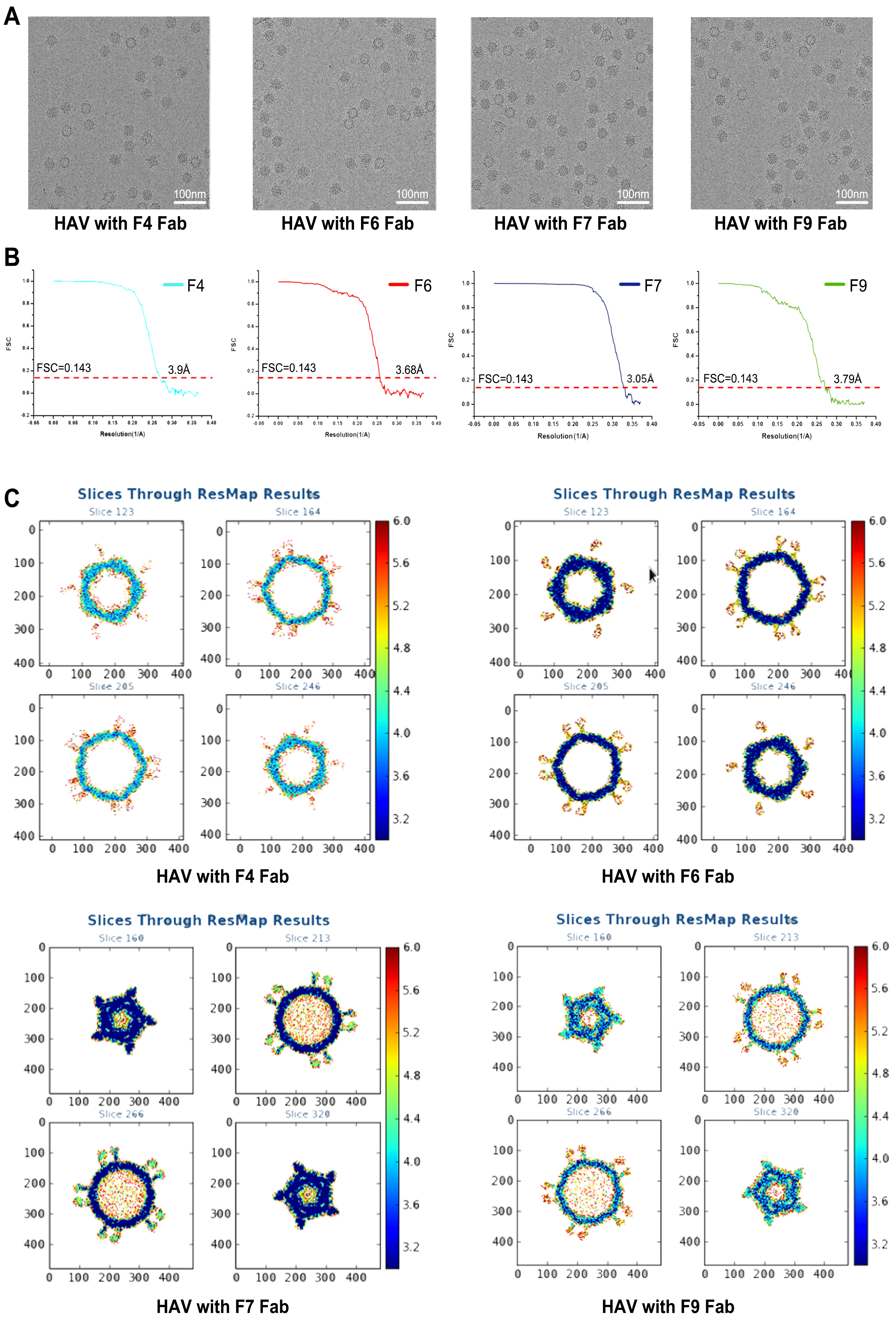

Supplement: S3 Fig — (A) Cryo-EM images of HAV particles complexed with F4, F6, F7, and F9 Fab. (B) The gold-standard FSC curves of complexes of F4 Fab-HAV, F6 Fab-HAV, F7 Fab-HAV, and F9 Fab-HAV. (C) Local resolution assessment. Local-resolution F4 Fab-HAV, F6 Fab-HAV, F7 Fab-HAV, and F9 Fab-HAV maps of density slices, rendered using ResMap [59], are shown. The red to blue color scheme corresponds to regions of relative low to high resolution. The underlying data of panel B can be found in S1 Data. cryo-EM, cryo-electron microscopy; Fab, fragment of antigen binding; FSC, fourier shell correlation; HAV, hepatitis A virus. (TIF) [file pbio.3000229.s003.tif]

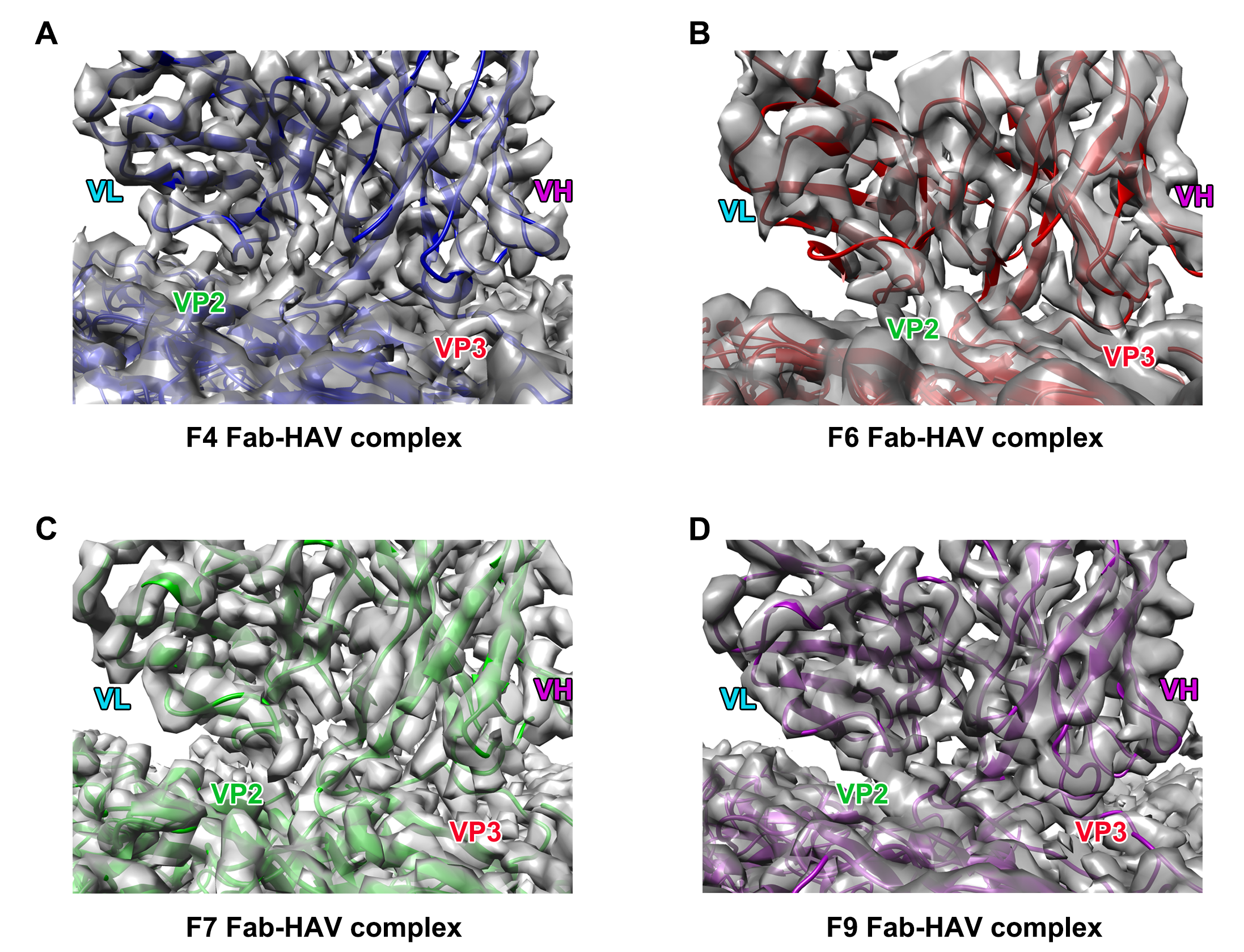

Supplement: S4 Fig — Closeup view of the interaction interface involving 4 of 6 CDRs on the Fab and surface capsids. F4 Fab-HAV, F6 Fab-HAV, F7 Fab-HAV, and F9 Fab-HAV are colored in blue, red, green, and purple in (A), (B), (C), and (D), respectively. CDR, complementary determining region; Fab, fragment of antigen binding; HAV, hepatitis A virus. (TIF) [file pbio.3000229.s004.tif]

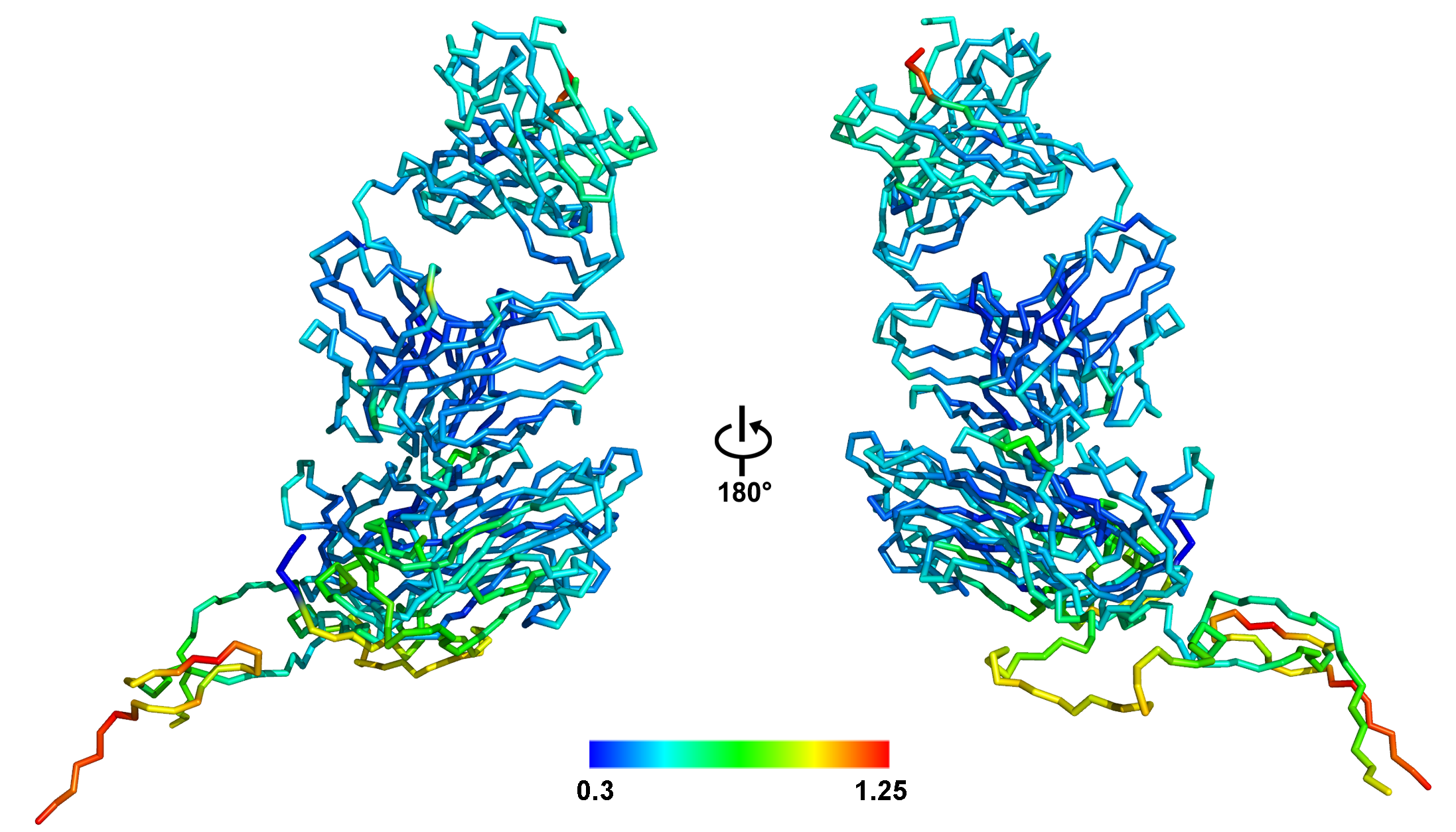

Supplement: S5 Fig — Structural comparisons of the 5 complexes—F4 Fab-HAV, F6 Fab-HAV, F7 Fab-HAV, F9 Fab-HAV, and R10 Fab-HAV—were superposed by pymol, and an asymmetry unit of Fab-HAV complex was colored by r.m.s.d. Fab, fragment of antigen binding; HAV, hepatitis A virus; r.m.s.d., root-mean-square deviation. (TIF) [file pbio.3000229.s005.tif]

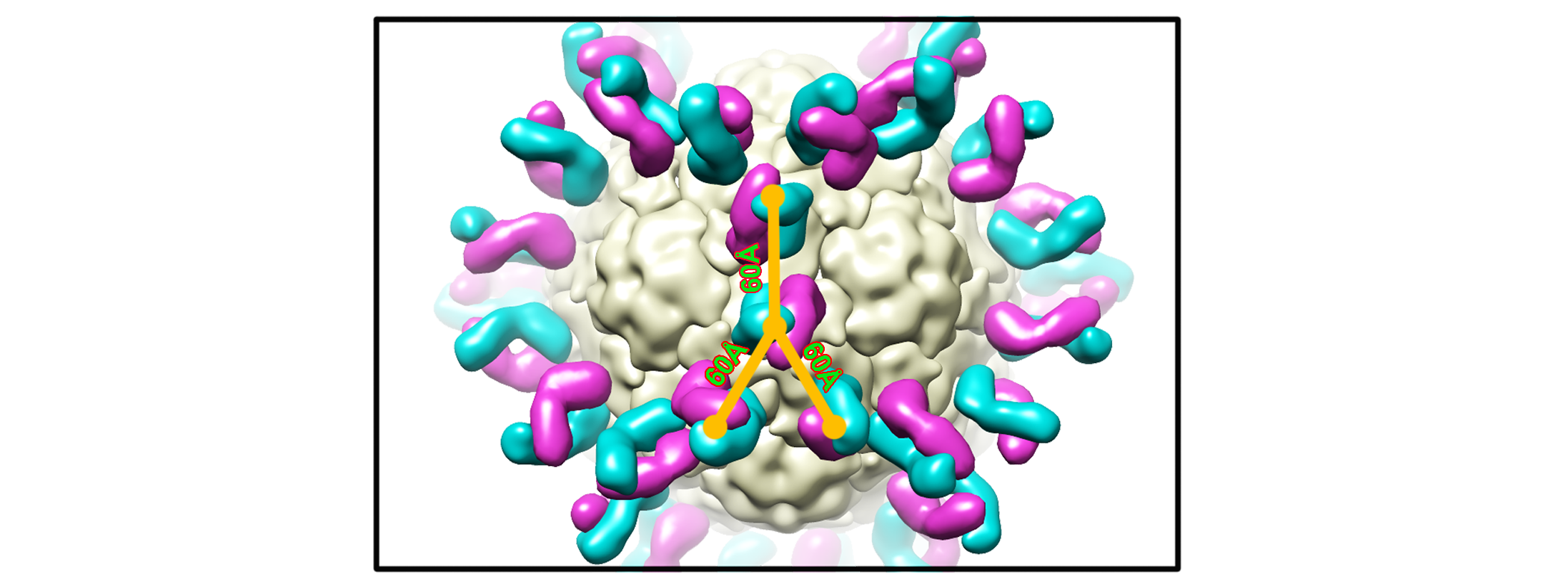

Supplement: S6 Fig — The distances between 2 adjacent Fabs are measured and labeled. Given the fact that the distance between 2 Fabs is approximately 60 Å, making low resolution surface from coordinates is done by Multi-scale Models in Chimera [60]. Fab, fragment of antigen binding. (TIF) [file pbio.3000229.s006.tif]

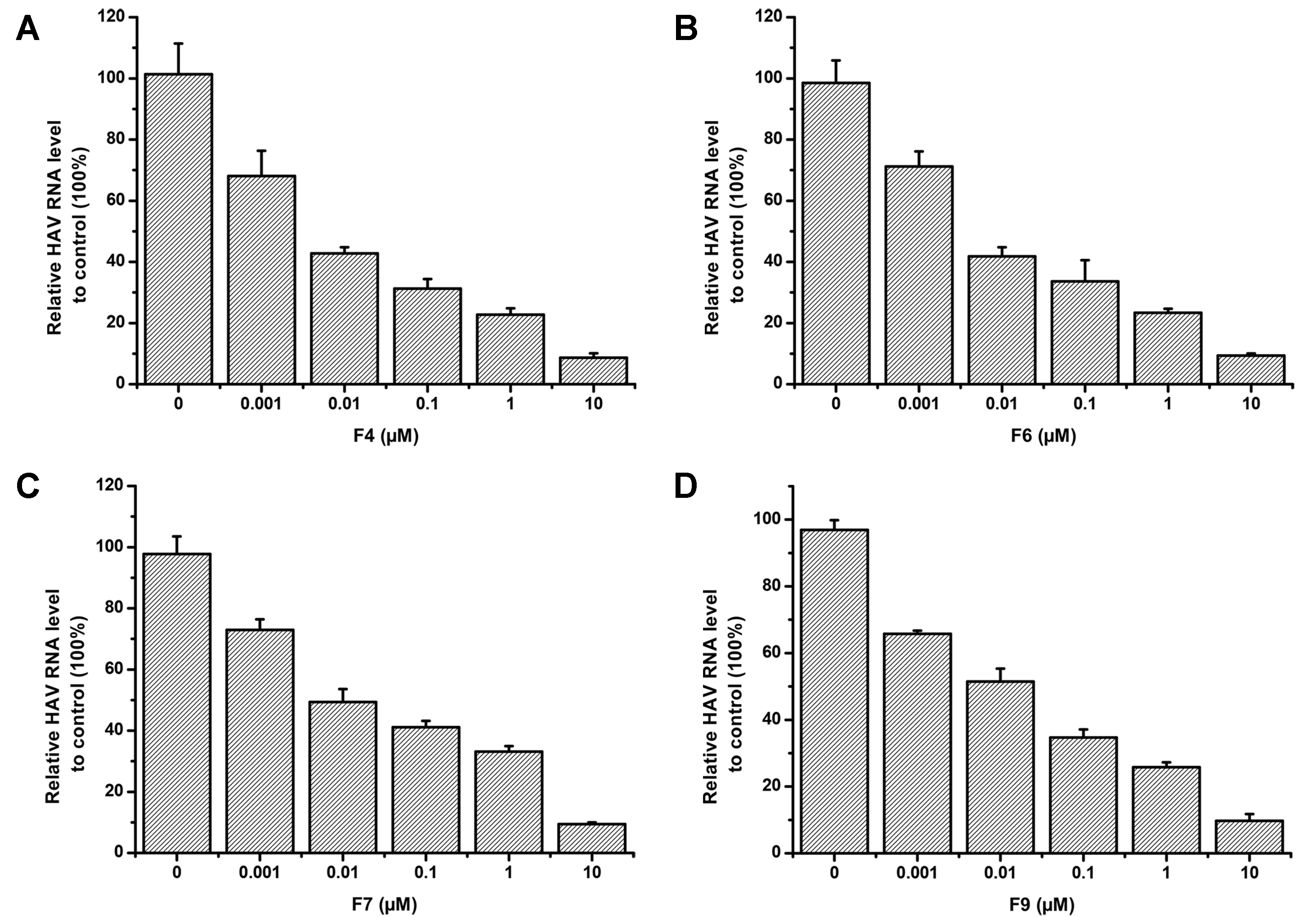

Supplement: S7 Fig — The amount of virions on the cell surface was detected by RT-PCR after binding to F4, F6, F7, or F9 before the virus was allowed to attach to 2BS cells. High concentrations of NAbs prevented attachment of HAV to the cell surface when HAV was exposed to antibodies before cell attachment. Data are presented as mean ± SD of 3 independent experiments. The underlying data of panels A to D can be found in S1 Data. HAV, hepatitis A virus; Fab, fragment of antigen binding; NAb, neutralizing monoclonal antibody; RT-PCR, reverse transcription PCR. (TIF) [file pbio.3000229.s007.tif]

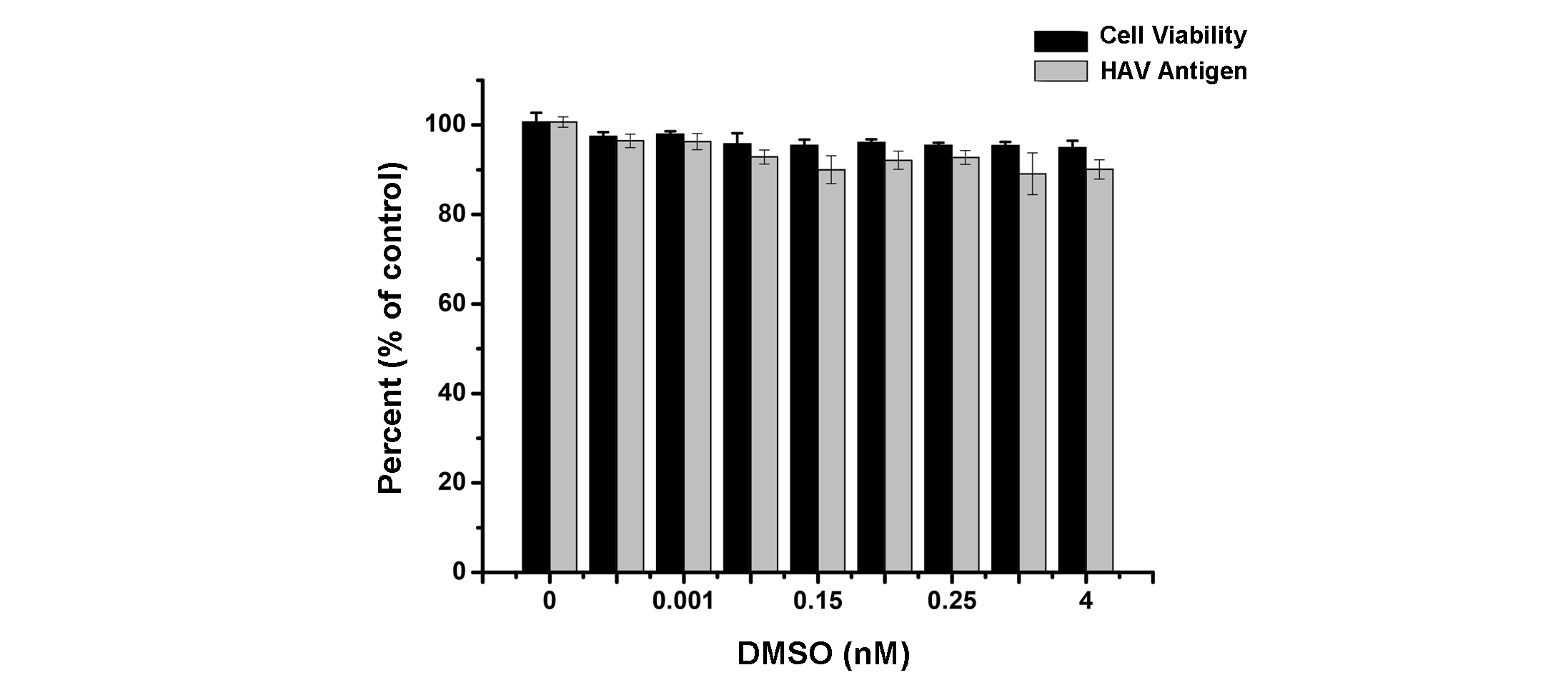

Supplement: S8 Fig — Various concentrations of DMSO were preincubated with HAV for 1 h at room temperature before infection of 2BS cells. The effects on virus titer were evaluated by determining HAV antigen content using indirect ELISA after 7 d of incubation. Values are mean ± SD. Experiments were repeated in triplicate. The assays of cytotoxic was determined by LDH release assay using the CCK-8 kit (Sangon Biotech, Shanghai) after 7 d of incubation. Data are presented as mean ± SD of 3 independent experiments. The underlying data of this figure can be found in S1 Data. CCK-8, cell counting kit-8; ELISA, enzyme-linked immunosorbent assay; HAV, hepatitis A virus; LDH, lactate dehydrogenase. (TIF) [file pbio.3000229.s008.tif]

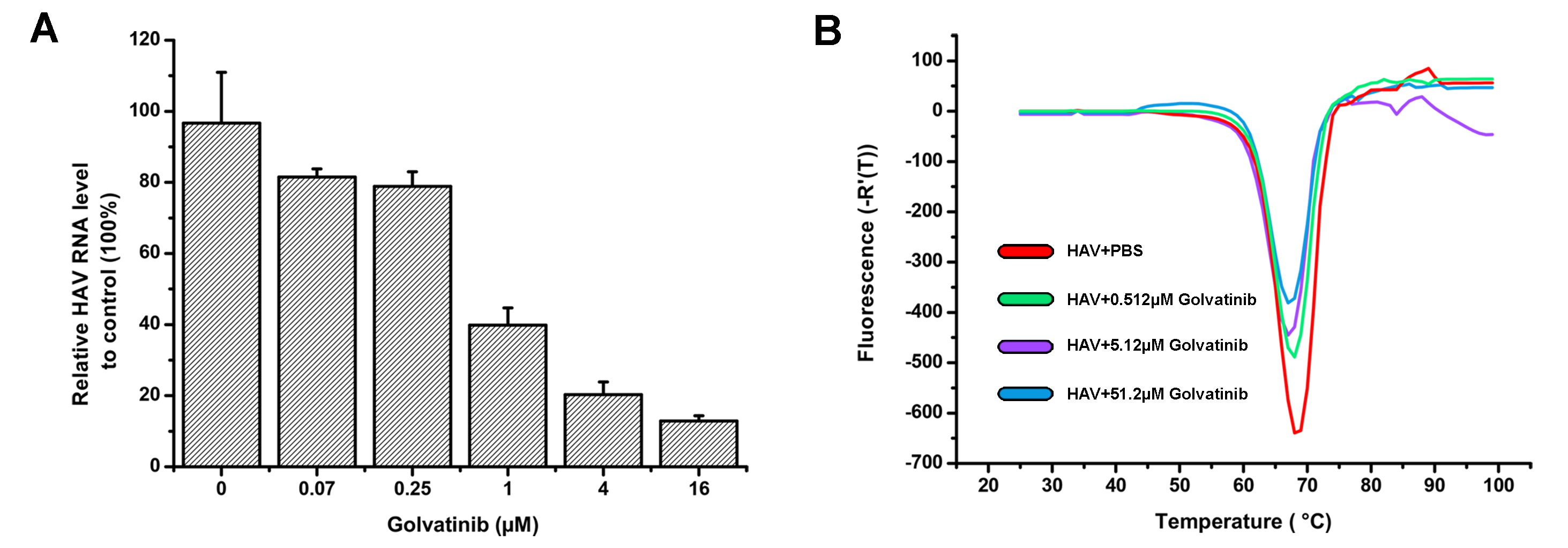

Supplement: S9 Fig — (A) Amount of virions on the cell surface was detected by RT-PCR after bind to golvatinib before the virus was allowed to attach to cells. Data are presented as mean ± SD of 3 independent experiments. (B) The stabilities of HAV particles in the presence of a serially diluted concentrations of golvatinib (0, 0.512, 5.12, and 51.2 μM) were determined by thermofluor assay using the dye SYTO9 to detect RNA exposure [57]. The binding of golvatinib to the HAV does not alter its particle stability, even in a highly concentrations of golvatinib. The underlying data of panels A and B can be found in S1 Data. HAV, hepatitis A virus; RT-PCR, reverse transcription PCR. (TIF) [file pbio.3000229.s009.tif]

**S6 Table**

| Compound | Structure | | Glide Score |
| --- | --- | --- | --- |
| 1 | 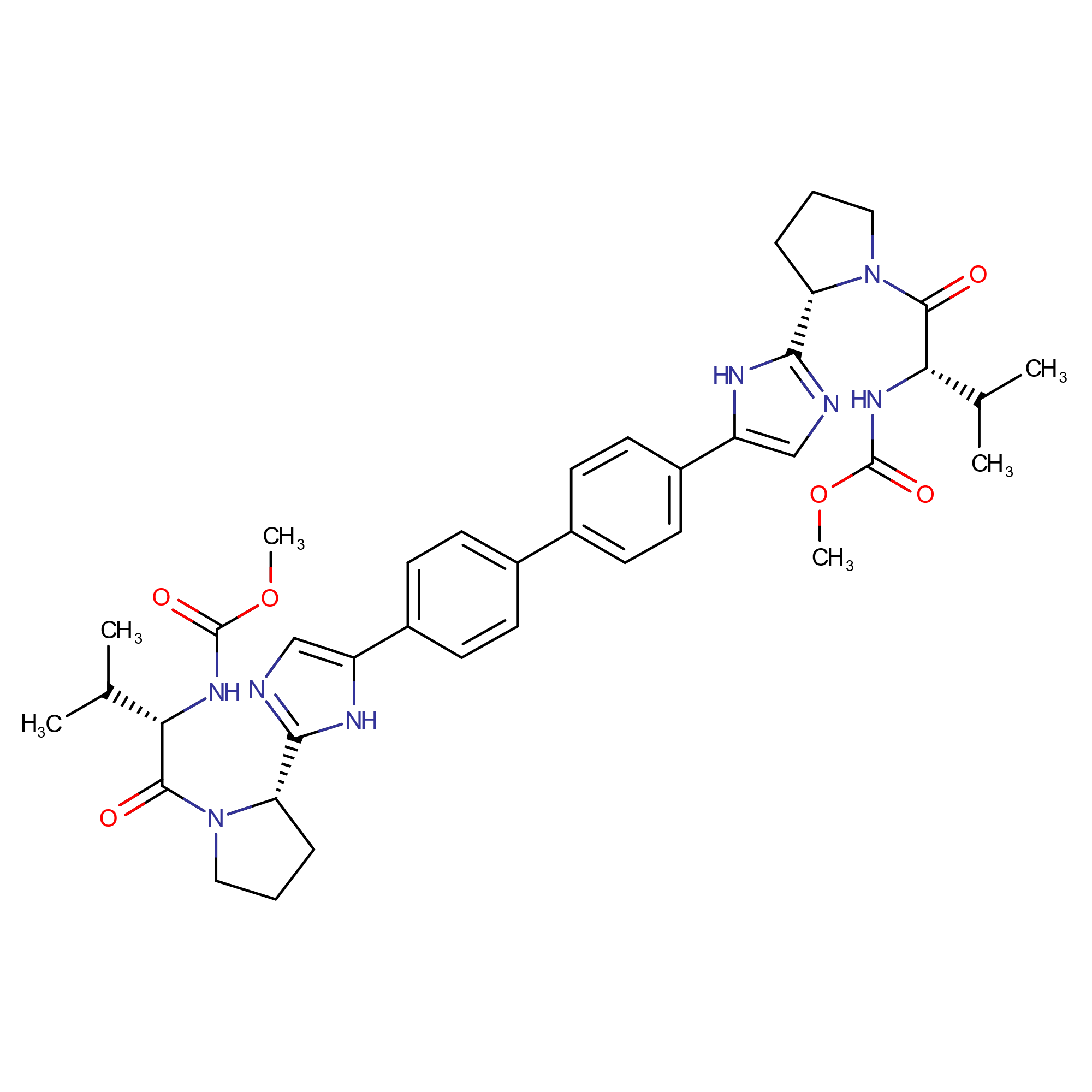 | -5.6 | |
| 2 | 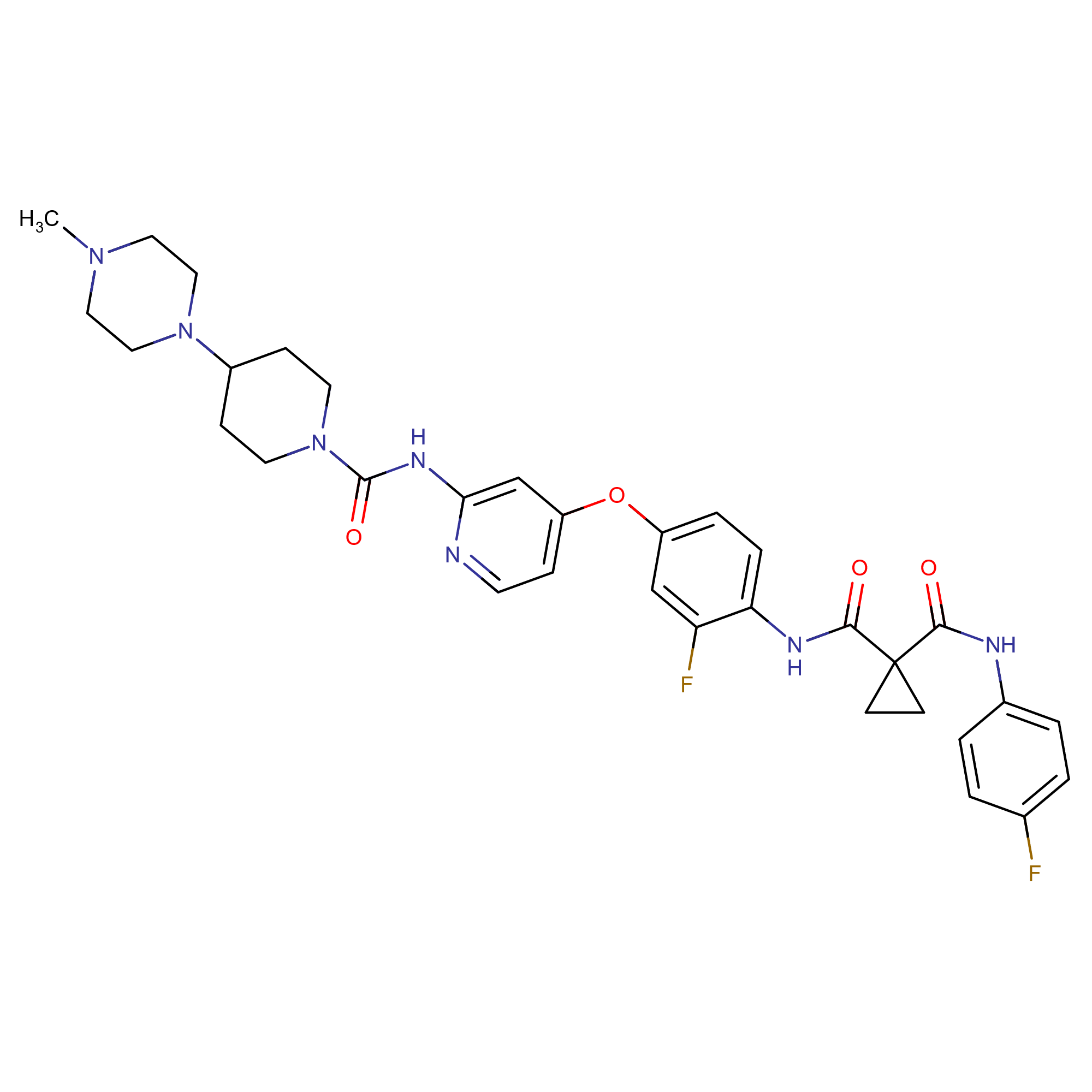 | -5.9 | |
| 3 | 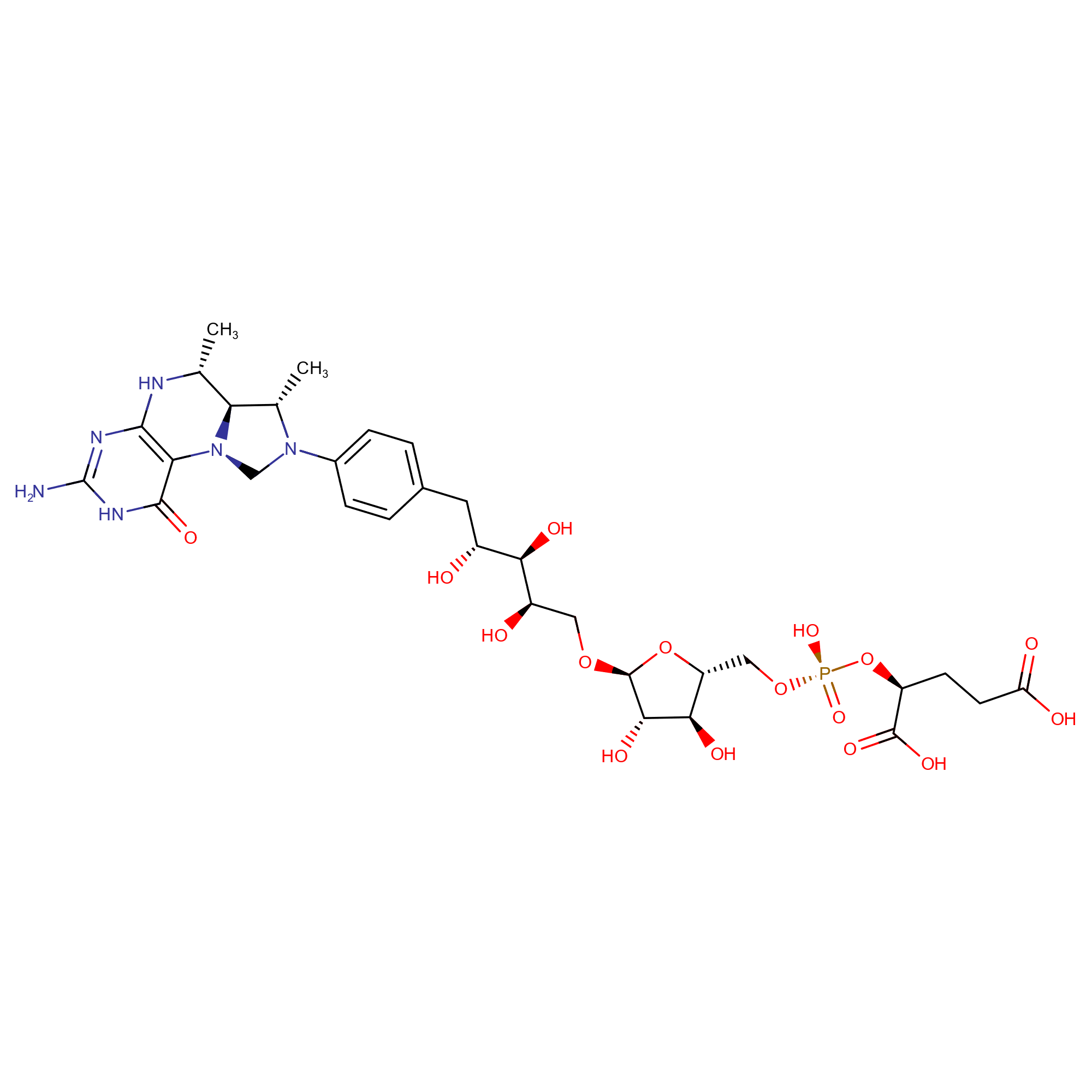 | -9.4 | |
| 4 | 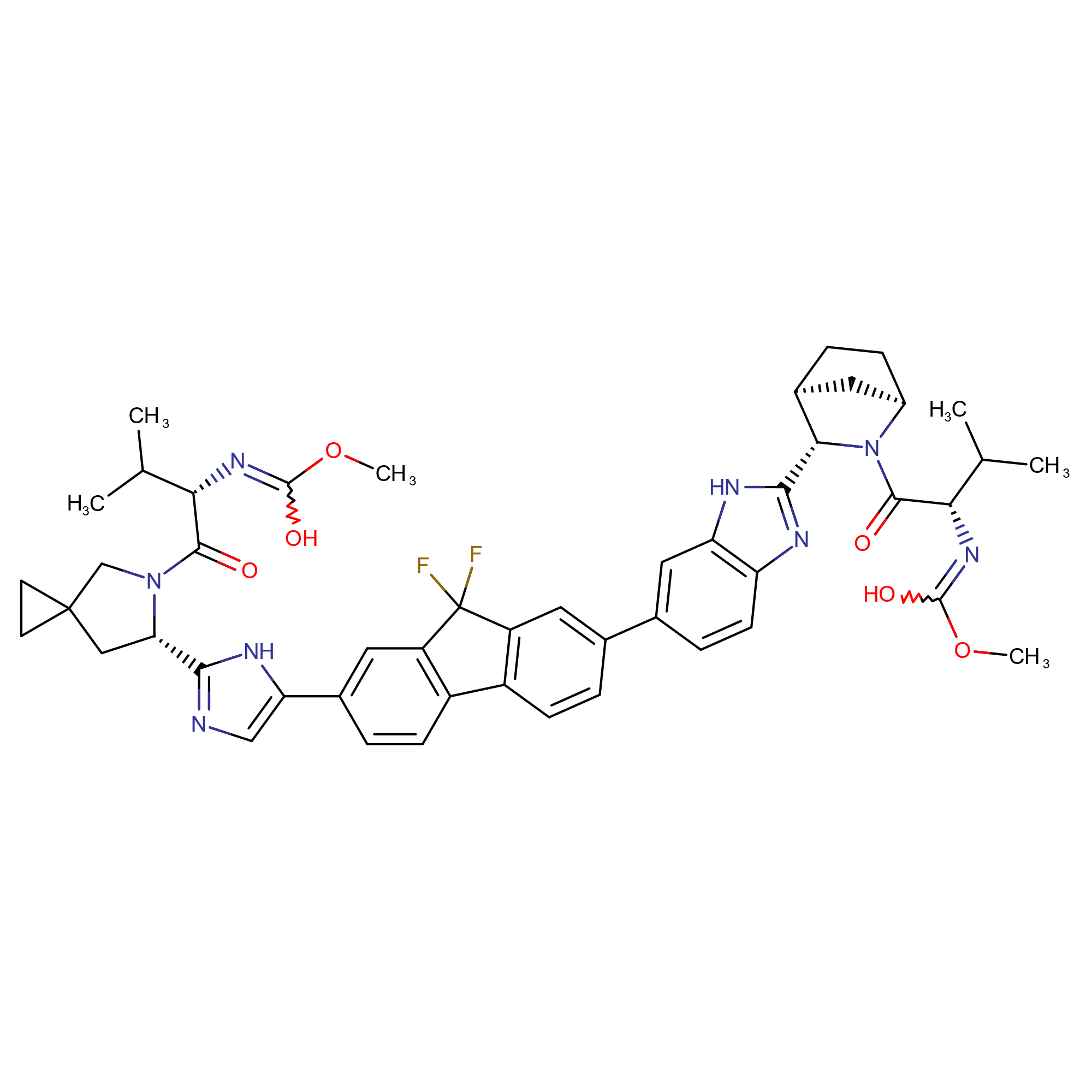 | -6.3 | |

Supplement: S6 Table — (DOCX) [file pbio.3000229.s015.docx]
