# Supplementary material for: Structural basis for neutralization of hepatitis A virus informs a rational design of highly potent inhibitors
Source: PLoS Biol. 2019 Apr 30;17(4):e3000229. doi: 10.1371/journal.pbio.3000229 (PMC6493668; doi:10.1371/journal.pbio.3000229)
Supplement: S3 Table — Fab, fragment of antigen binding; HAV, hepatitis A virus. (DOCX) [file pbio.3000229.s012.docx]

**S3 Table**

| L-chain | A-VP2 | B-VP3 | H-chain | A-VP2 | B-VP3 |
| --- | --- | --- | --- | --- | --- |
| Y31 |  | K150 | T28 |  | Q246 |
| R45 |  | S69 | N30 |  | T145 |
|  |  | S71 |  |  | Q246 |
| Y48 |  | S71 | C31 |  | Q75 |
|  |  | V72 |  |  | V78 |
| S51 | R67 |  |  |  | T145 |
| R52 | S65 | V72 | Y32 |  | Q74 |
|  | R67 | R209 |  |  | Q75 |
|  | S201 |  | T52 |  | D143 |
| L53 | R67 | R209 |  |  | T145 |
| F55 |  | A68 | A53 |  | T145 |
|  |  | S69 | A54 |  | D143 |
|  |  | R209 | S56 |  | D143 |
| T59 | T71 |  | Y57 |  | D143 |
|  | A198 |  | R98 |  | Q74 |
|  |  |  | N101 |  | S71 |
|  |  |  |  |  | V72 |
|  |  |  |  |  | G73 |
|  |  |  |  |  | Q74 |
|  |  |  |  |  | T145 |
|  |  |  |  |  | I147 |
|  |  |  |  |  | T148 |
|  |  |  |  |  | L149 |
|  |  |  | V102 |  | V72 |
|  |  |  |  |  | T148 |
|  |  |  |  |  | L149 |
|  |  |  | T103 | S65(2) | V72 |
|  |  |  |  |  | T148 |
|  |  |  |  |  | L149 |
|  |  |  |  |  | K150 |
|  |  |  | Al04 |  | T148 |
|  |  |  |  |  | K150 |
|  |  |  | Q105 |  | T148 |
|  |  |  | Y106 |  | G146 |
|  |  |  |  |  | T148 |
