# Supplementary material for: Structural basis for neutralization of hepatitis A virus informs a rational design of highly potent inhibitors
Source: PLoS Biol. 2019 Apr 30;17(4):e3000229. doi: 10.1371/journal.pbio.3000229 (PMC6493668; doi:10.1371/journal.pbio.3000229)
Supplement: S5 Table — NAb, neutralizing monoclonal antibody. (DOCX) [file pbio.3000229.s014.docx]

**S5 Table**

| Complex | Binding energy(kcal/mol) | | Interface Area(Å^2^) | neut_50_(nM) |
| --- | --- | --- | --- | --- |
| F4 Fab-HAV | -76.71±8.86 | 1232.2 | | 0.25 |
| F6 Fab-HAV | -100.88±10.70 | 1288.1 | | 0.13 |
| F7 Fab-HAV | -45.56±5.73 | 1168.1 | | 0.42 |
| F9 Fab-HAV | -26.44±6.35 | 1156.3 | | 0.85 |
| R10 Fab-HAV | -12.34±5.32 | 966.7 | | 2.03 |
